# Large-Scale Evolutionary Analyses on SecB Subunits of Bacterial Sec System

**DOI:** 10.1371/journal.pone.0120417

**Published:** 2015-03-16

**Authors:** Shaomin Yan, Guang Wu

**Affiliations:** State Key Laboratory of Non-food Biomass Enzyme Technology, National Engineering Research Center for Non-food Biorefinery, Guangxi Biomass Industrialization Engineering Institute, Guangxi Key Laboratory of Biorefinery, Guangxi Academy of Sciences, 98 Daling Road, Nanning, Guangxi, 530007, China; University of Illinois at Chicago College of Medicine, UNITED STATES

## Abstract

Protein secretion systems are extremely important in bacteria because they are involved in many fundamental cellular processes. Of the various secretion systems, the Sec system is composed of seven different subunits in bacteria, and subunit SecB brings secreted preproteins to subunit SecA, which with SecYEG and SecDF forms a complex for the translocation of secreted preproteins through the inner membrane. Because of the wide existence of Sec system across bacteria, eukaryota, and archaea, each subunit of the Sec system has a complicated evolutionary relationship. Until very recently, 5,162 SecB sequences have been documented in UniProtKB, however no phylogenetic study has been conducted on a large sampling of SecBs from bacterial Sec secretion system, and no statistical study has been conducted on such size of SecBs in order to exhaustively investigate their variances of pairwise p-distance along taxonomic lineage from kingdom to phylum, to class, to order, to family, to genus and to organism. To fill in these knowledge gaps, 3,813 bacterial SecB sequences with full taxonomic lineage from kingdom to organism covering 4 phyla, 11 classes, 41 orders, 82 families, 269 genera, and 3,744 organisms were studied. Phylogenetic analysis revealed how the SecBs evolved without compromising their function with examples of 3-D structure comparison of two SecBs from *Proteobacteria*, and possible factors that affected the SecB evolution were considered. The average pairwise p-distances showed that the variance varied greatly in each taxonomic group. Finally, the variance was further partitioned into inter- and intra-clan variances, which could correspond to vertical and horizontal gene transfers, with relevance for *Achromobacter*, *Brevundimonas*, *Ochrobactrum*, and *Pseudoxanthomonas*.

## Introduction

All living cells have the capacity to exchange material and information with their surrounding environment. Given that one third or one quarter of the bacterial proteins work either in the cell envelope or outside of the cell [1], the materials are exchanged through various transport systems imbedded in the cell membrane, and the information is exchanged through various receptors on the cell surface. The secretion of proteins that are synthesized inside the cells into the extracellular space is essential for the survival of cells, and its importance is connected with the increasing number of cellular functions such as cell division, membrane biogenesis, motility, etc. [2]. On the other hand, the protein secretion system is very different from the transport systems that transport water, inorganic ions, and small organic molecules because a cell can purposely synthesize different proteins, whose sizes and conformations can vary one from another. Because of this importance, the protein secretion system has been the focus of many studies, and is named in various ways at different times as seen in various databases and literature such as export membrane protein, protein-export membrane protein, preprotein translocase, protein translocase, secretion system, secretory pathway, translocase nanomachine, translocon, transmembrane export protein, etc. For such an important cellular system, it is not yet known as to when it first appeared in cells, and thereafter how it has evolved up to the present time.

Over the evolution, archaea, bacteria, and eukaryota have developed different secretion systems because of their different architectures. However, at least one secretion system comes into sight across all these three superkingdoms, that is, the Sec system, which was previously termed as general secretory pathway (GSP) [3, 4]. Actually, the difference in cellular architecture exists not only across superkingdoms but also within kingdom. For example, the cell membrane is simpler in Gram-positive bacteria than in Gram-negative bacteria. So far the secretion systems have been classified into six types in Gram-positive bacteria [5–7], and seven types in Gram-negative bacteria [6, 8], where the Sec system belongs to the type II secretion system [2, 4].

For various reasons, the secretion systems in Gram-negative bacteria have been studied in great details [9], so the knowledge on the type II secretion system is relatively rich. The type II secretion system applies two steps to secrete proteins into extracellular space, (i) proteins are moved across the inner membrane of the cell through the Sec system and then (ii) they are moved across the outer membrane of the cell [3, 4]. Actually, the importance of the Sec system is further emphasized by the fact that the largest amount of predicted secreted proteins was estimated to pass through the Sec system [10].

The Sec system in bacteria is composed of seven subunits, i.e. SecA, SecB, SecD, SecE, SecF, SecG and SecY [11]. SecY, SecE and SecG work together as the SecYEG complex, which forms a protein-conducting channel across the inner membrane. It was found that SecG was not necessarily essential but increased the translocation efficiency [12–14]. SecD and SecF function together as the SecDF complex, which promotes the final stage of translocation of secreted proteins through the inner membrane. As an ATP-dependent motor, SecA with SecYEG and SecDF forms a complex associated with the cytoplasmic membrane, driving the stepwise translocation of secreted proteins. Although SecB is not associated with the cytoplasmic membrane, it brings precursor proteins to SecA [15, 16], so SecB seems simple but is very specific and different from the other six subunits.

The subunits in the eukaryotic Sec system have different names, and include Sec12, Sec13, Sec16, Sec23, Sec24, Sec31 and Sec61. Sec12 at the endoplasmic reticulum is somewhat similar to SecA [12, 17, 18], but it uses GTP rather than ATP [19]. Sec23, Sec24, Sec13 and Sec31 together with Sar1 form the coat protein complex II (COPII) coat machinery [20]. Sec16 is apparently not directly required for vesicle formation [21]. To some extent, Sec24 in eukaryota could be somewhat parallel to SecB in bacteria. Sec24 is thought to be the primary subunit that binds to the secreted proteins at the endoplasmic reticulum and then concentrates them into the forming vesicle [22], because cargo-binding pockets and their corresponding signal sequences were identified in Sec24, termed as A-, B- and C-sites [23]. Sec61 corresponds to the SecYEG complex [24]. Moreover, recent studies have suggested that Sarl also plays a role in cargo recognition by binding to the export signals [25].

In archaea, the secretion systems were largely studied through genomic sequencing data, and then compared with those of bacteria and eukaryota [26, 27], which showed clear similarities in the Sec system among bacteria, eukaryota and archaea [28]. For example, Mj0357 protein from *Methanococcus jannaschii* has 18% sequence identical to SecB from *Escherichia coli*, but its physicochemical properties are highly similar to those of *Escherichia coli* SecB [29]. On the other hand, SecA is absent from archaea while SecY is very similar between archaea and bacteria.

As abovementioned, the function of SecB appears relatively simple but specific, i.e. to bring the secreted protein to SecA, however there are several names to demonstrate its importance such as the cytosolic chaperone SecB, the export-specific chaperone SecB, etc. In fact, the binding of SecB to SecA in the cytosol has a low affinity, whereas SecB has a high affinity to bind to the SecYEG-bound SecA [30]. Another function that SecB performs is the antifolding of preprotein [31]. Naturally, the structure of SecB is characterized with two portions, one to bind to the secreted protein and another to bind to the SecA [32]. The beta-structure prevails in SecB, and the secreted proteins may recognize SecB through beta-beta interaction. Actually, what SecB carries are the unfolded preproteins [33, 34], which are co-translational translocation in eukaryota but post-translation translocation in bacteria [35]. Likely, SecB has no preference to a specific structure of preproteins [36]. The association of SecB with SecA occurs between the 13 amino acid residues of a negative charged ring on the beta-sheet of SecB [34, 37, 38] and the last 22 amino acid residues at the carboxyl terminus of the SecYEG-bound SecA [39, 40]. Their binding stimulates the ATPase activity in SecA [22], and then allows for an initial insertion of a loop-like structure comprising the signal sequence and the early mature domain of the preprotein into the SecYEG channel, and thereafter SecB is dissociated from SecA [41, 42].

**Fig 1:**
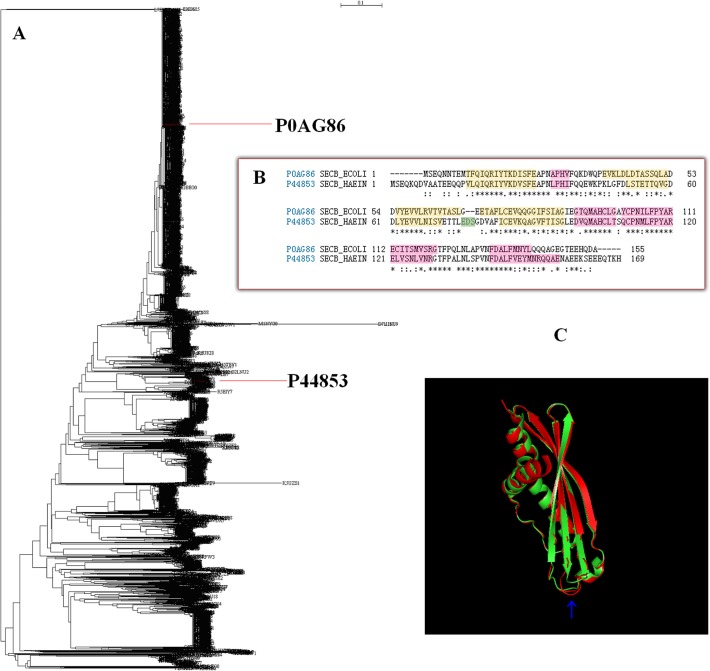
Locations of SecB P0AG86 from *Escherichia coli* and SecB P44853 from *Haemophilus influenzae* in phylogenetic tree composed of 3813 bacterial SecBs (A), amino acid sequence alignment of SecB P0AG86 and SecB P44853 (B), and 3-D structure alignment of SecB P0AG86 (PDB accession number 1QYN) in green color and SecB P44853 (PDB accession number 1FX3) in red color (C). The blue arrow shows the amino acid turn in SecB P44853.

It is suggested that SecB should have evolved at the very early stages of life and should widely exist because of its function as a carrier. The Sec secretion system would work poorly if one of its subunits SecA, SecD, SecE, SecF, SecG, and SecY functions poorly but it would hardly work without SecB to bring secreted proteins to the Sec secretion system. Therefore, it is absolutely necessary to study the evolutionary relationship of subunit SecB from bacterial translocase nanomachine. It is important to note that SecB functions in the form of tetramer composed of two dimmers, which further form two long channels along the side of the molecule. These are conserved hydrophobic amino acids in flexible loops, providing a suitable environment to bind preproteins, while there is an acidic region on the top surface for binding to SecA [43]. Currently, several 3-D structures of SecB have been documented in the PBD [44], of which two are not in complex associated SecA. The comparison indicates that the difference between these two SecB sequences is over a half (Fig. 1B), however there is little difference between their 3-D structures (Fig. 1C), which guarantee the function of SecB. This is a big mystery of evolution, i.e. SecBs have evolved under different selective pressures with uncountable factors but they keep working well in bacteria. This issue can now be addressed together with the help of phylogenetic and statistical analyses, as 5162 SecB sequences had been documented in UniProtKB [45] until February 2014, and give the possibility to conduct a large-scale phylogenetic analysis. With this huge amount of data, it is equally interesting to conduct a detailed statistical analysis on each taxonomic group. Although SecBs exist so widely, their statistical characteristic is unclear in each taxonomic group. Technically, this requires the further elaboration of 5162 SecB sequences in order to track each SecB along the taxonomic lineage from phylum to class, to order, to family, to genus and finally to organism, which brings about 3813 SecB sequences with fully and clearly documented taxonomic lineage. The aim of this study was designed to analyze these 3813 SecB sequences phylogenetically and statistically.

## Results and Discussion

Fig. 1 demonstrates the comparison between two SecB sequences (accession numbers P0AG86 and P44853). The exact evolutionary locations of SecBs P0AG86 and P44853 are labeled in full phylogenetic tree (Fig. 1A), and their independent evolution can be witnessed. The SecB P0AG86 came from *Escherichia coli* (strain K12) belonging to the phylum *Proteobacteria*, class *Gammaproteobacteria*, order *Enterobacteriales*, family *Enterobacteriaceae* and genus *Escherichia* [15, 46], while the SecB P44853 was from *Haemophilus influenzae* (strain ATCC 51907/DSM 11121/KW20/Rd) belonging to the phylum *Proteobacteria*, class *Gammaproteobacteria*, order *Pasteurellales*, family *Pasteurellaceae* and genus *Haemophilus* [46]. In Fig. 1A, the SecB P0AG86 underwent a longer evolution period than the SecB P44853, since the SecB P0AG86 had 50 bifurcations while the SecB P44853 had only 32 ones. Meanwhile, they did not belong to sister clans, which raised the possibility that genus *Escherichia* had evolved independently several times, and its turnover of genetic materials could be sufficiently high. Indeed, the SecB P0AG86 became shorter and lacked of the amino acid turn that existed in the SecB P44853, which is marked as the residues in yellow color in Fig. 1B and pointed with a blue arrow in Fig. 1C. The genetic materials in the SecB P0AG86 were lost through a 50-step evolution, because their common ancestor seemed to possess this amino acid turn. However, this loss did not result in a significant change in their 3-D structure, although their identical amino acids account only for 48.521%. Indeed, 43% of the surface of SecB was covered by the 41 positions [47], which was unlikely to contain the amino acid turn. In fact, the buried cysteine in SecB, which should be involved in the stabilizing interactions at the dimer interface [48], was crucial for tight packing, because mutations were likely to disturb the tetramer formation but not the dimer formation [46].

The 3-D structures of SecBs P0AG86 and P44853 are valuable not only because they are the only two structures not in complex form in the PDB but also because they can throw light on the evolution mechanisms of SecB. At first, SecB interacted with a long nascent polypeptide chain of secreted preprotein [49], which had yet to begin its folding [33, 34], and therefore SecB had no preference to a specific structure of preproteins [36]. As a consequence, the amino acid turn in the SecB sequence appeared not to be absolutely necessary, so the evolution squeezed these amino acids out of the SecB sequence when comparing the SecB P0AG86 with the SecB P44853. This observation can be supported by the finding that SecB and the nascent chain interaction was independent of the presence of a signal sequence in preprotein [50]. Except for the amino acid turn in the SecB sequence, the other structures were necessarily essential because the translocation of some preproteins, whose signal sequences had been removed, strictly required SecB [51, 52]. An experimental study also revealed that the crossover loop and the helix-connecting loop were part of the SecB substrate-binding site and that SecB could regulate the access to substrate-binding site by modulating the conformation of these regions [46]. Therefore Fig. 1 demonstrates how evolution conserves the key elements, but it is unclear whether this evolution led to subfunctionalization of SecB in different species.

**Fig 2:**
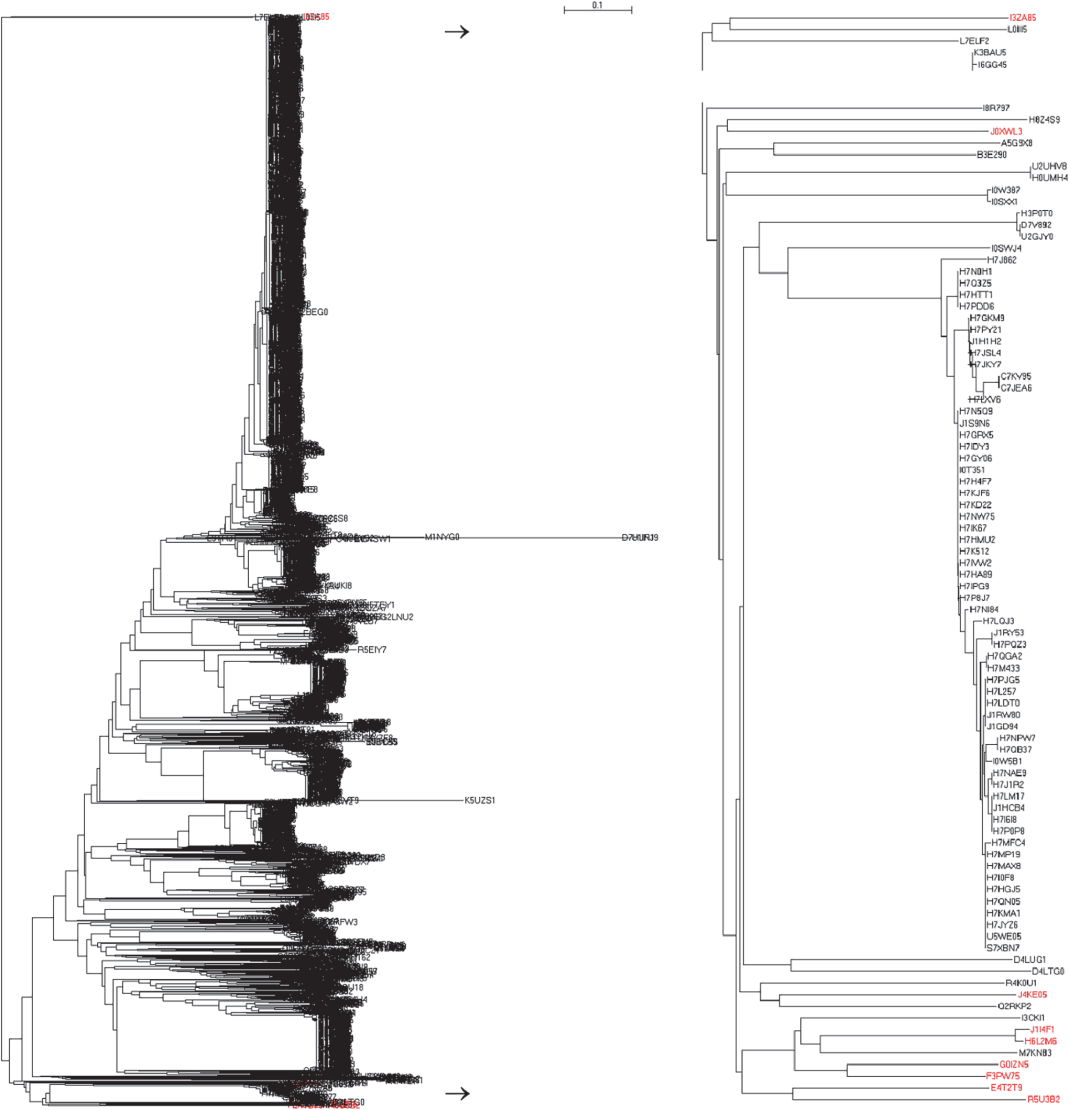
Distribute of SecBs from phylum *Bacteroidetes* in full phylogenetic tree of 3813 bacterial SecBs (left-hand column) with their detail distribution (right-hand column). The red colored branches are SecBs from phylum *Bacteroidetes*. The detailed phylogenetic tree in Newick tree format with maximum likelihood bootstrap values on all branches is available in Supplementary Materials.

**Fig 3:**
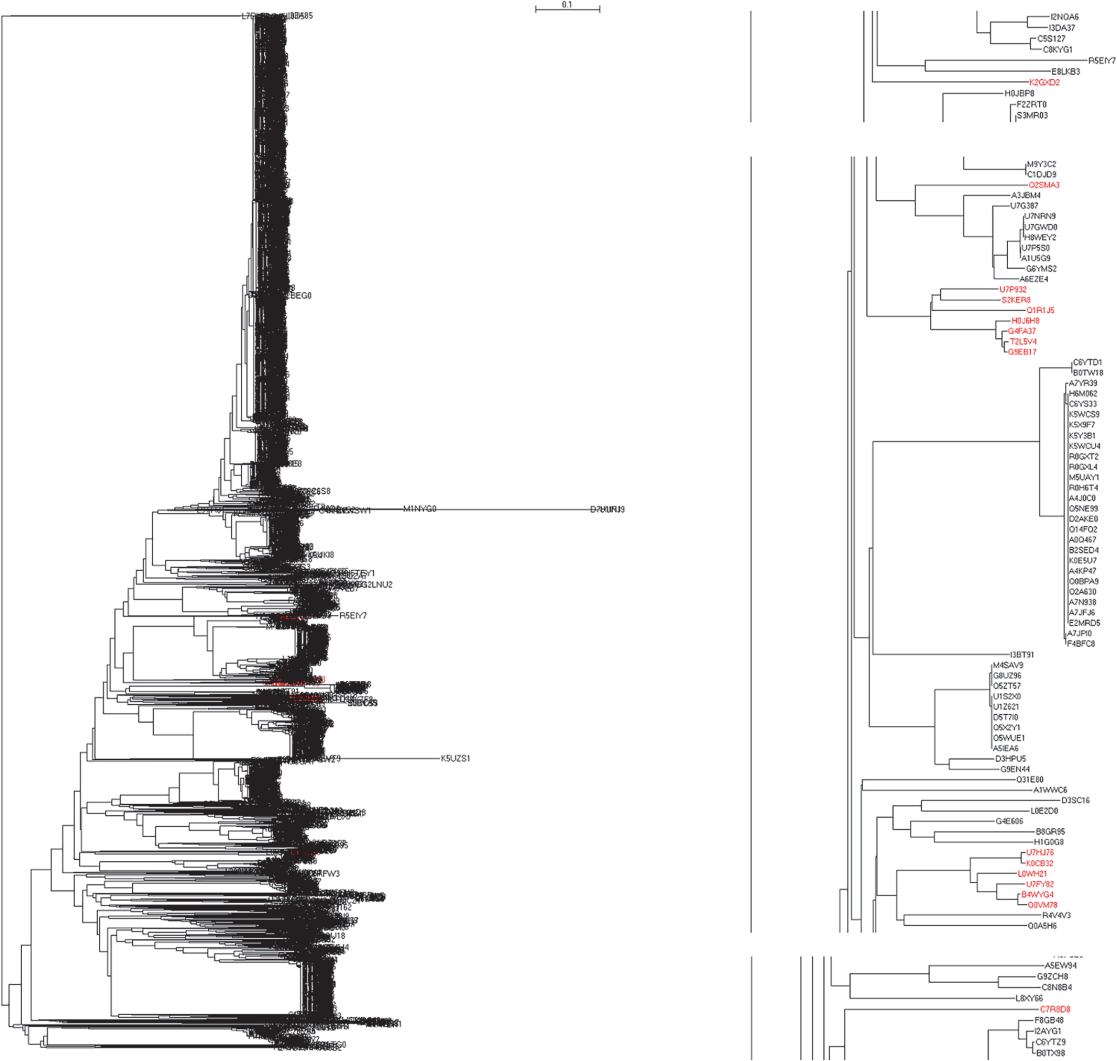
Location of SecBs from order *Oceanospirillales* in full phylogenetic tree of 3813 bacterial SecBs (left-hand column) and in portions of phylogenetic tree (right-hand column). The accession numbers in red color are SecBs from order *Oceanospirillales*. The detailed phylogenetic tree in Newick tree format with maximum likelihood bootstrap values on all branches is available in Supplementary Materials.

Fig. 2 shows the complete phylogenetic tree of 3813 bacterial SecBs in the left-hand panel (the detailed phylogenetic tree with bootstrap value and branch length in Newick tree format is available in Supplementary Materials), where their evolution can be traced. For a simple example, nine SecBs came from the phylum *Bacteroidetes* (marked in red color), which distributed themselves both at the top and the bottom of the phylogenetic tree (right-hand panel). Seven of them were clustered together at the bottom with the SecBs M7KN83 and I3CKI1 from the phylum *Proteobacteria* and the SecBs Q2RKP2 and R4K0U1 from the phylum *Firmicutes*, while the SecB J0XWL3 from the phylum *Bacteroidetes* was located near the bottom of the phylogenetic tree with the SecBs H8Z4S9, A5G9X8 and B3E290 from the phylum *Proteobacteria* and the SecB I8R797 from the phylum *Firmicutes*. However, the SecB I3ZA85 from the phylum *Bacteroidetes* was located at the top of the phylogenetic tree with the SecBs L0III5 and L7ELF2 from the phylum *Firmicutes*. Also, there were 16 SecBs from the phylum *Proteobacteria*, class *Gammaproteobacteria* and order *Oceanospirillales*, located in the middle of the phylogenetic tree in Fig. 3, of which eight, one and seven belong to the families *Alcanivoracaceae*, *Hahellaceae* and *Halomonadaceae*, respectively. The SecBs from the family *Alcanivoracaceae* and genus *Alcanivorax* formed a cluster, except for the SecB K2GXD2 that evolved near the SecBs E8LKB3 and R5EIY7 from the order *Aeromonadales*, family *Succinivibrionaceae* and genus *Succinatimonas*, whereas the SecB C7R8D8 from the genus *Kangiella* evolved near the SecBs from the order *Thiotrichales*, family *Francisellaceae* and genus *Francisella*. Seven SecBs from the family *Halomonadaceae* built another cluster, and the SecB Q2SMA3 from the family *Hahellaceae* evolved closely with nine SecBs from the order *Alteromonadales*, family *Alteromonadaceae* and genus *Marinobacter*.

The abovementioned examples are very suggestive, because *Proteobacteria* has a different mechanism to secrete proteins via the Sec secretion system. The secreted proteins lacked of classical signal peptides, so the mechanism was SecA-dependent but SecB-independent [53] although SecB was unique to *Proteobacteria* [54]. However, the phylogenetic tree implied that they had evolved from a common ancestor, which is in good agreement with the Darwinian theory [55], and since then they had evolved multiple clans at multiple times. Another possible explanation could be the temperature, which had a great influence on the Sec secretion system, especially SecB [56–58]. For example, SecB was required for *Escherichia coli* to grow at low temperature [53, 59]. Interestingly enough, the bacteria from the phylum *Proteobacteria*, class *Gammaproteobacteria* and order *Oceanospirillales* played a great role in eating the oil spilled into the Gulf of Mexico in 2010 [60]. Likely, the water temperature as well as the salt condition [61] could be factors promoting the development of the abovementioned different mechanisms.

Nevertheless, many interesting issues related to SecB evolution can be dug out from the phylogenetic tree in view of different aspects, such as SecBs in *Serratia marcescens*, which was intensively studied [31, 62–64] and also included in the phylogenetic tree. However, the analysis on phylogenetic tree has to stop here due to the limit of space.

**Fig 4:**
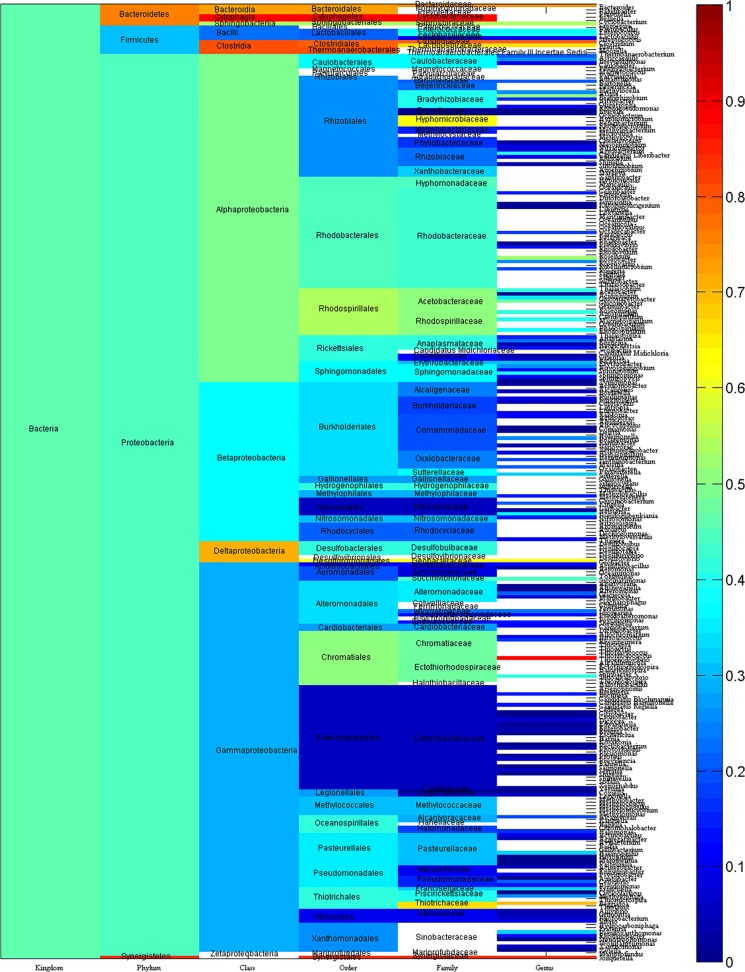
Average pairwise p-distance of bacterial SecB. Blank: average pairwise p-distance is not unavailable.

Fig. 4 displays the average pairwise p-distance of SecB sequences in each taxonomic group from kingdom to phylum, to class, to order, to family and finally to genus. For bacterial SecBs, the average p-distance was 0.4546, which was the mean value of p-distances for each of the 3813 SecBs versus the rest of the 3812 SecBs. This value varied greatly, indicating the divergence of bacterial SecBs in each taxonomic group. For example, the average p-distance was statistically larger in the phylum *Proteobacteria* than in the phylum *Firmicutes* (0.4390±0.1429, n = 3727 vs 0.3087±0.2392, n = 75, mean±SD, *p* < 0.001) in Fig. 4. Nevertheless, the phylum *Proteobacteria* had more organisms than the phylum *Firmicutes* did; a similar phenomenon was observed long ago [55], namely, the larger a taxonomic group is, the more variants it has. It is interesting to note that the SecBs in the phylum *Synergistetes* were colored in red, which was very different from other phyla, suggesting that the evolution of *Synergistetes* was very different from the rest phyla, otherwise the phylum *Synergistetes* would not be so different. This is plausible because *Synergistetes* is a phylum that was recognized only recently. Its organisms lived in animal gastrointestinal tracts, soil, oil wells and wastewater treatment plants, so they belonged to anaerobic bacteria that had a rod/vibrioid cell shape with Gram-negative staining [65, 66]. The experiments did not detect the genes for various proteins that involved in the lipopolysaccharides biosynthesis in *Synergistetes* although they had a diderm cell envelope, thus they might have an atypical outer cell envelope [67, 68]. This diversity could have resulted from multiple linear motifs that either lost or gained during the evolution [69], and may serve as a typical example of the evolution in multiple clans at multiple times.

So far the best-studied organism that possesses SecB is *Escherichia coli*, and 993 SecB sequences from *Escherichia coli* were included in this study. Their average p-distance was really very small (0.0052±0.0265), while their average number of amino acids was 154.93±1.29, so the alignment of any two SecB sequences from *Escherichia coli* would have only one amino acid different from the other. Perhaps this is one of reasons of why the subunits from the Sec system of *Escherichia coli* are often expressed in other species. Indeed, *Bacillus subtilis* and its sister clans are widely used to produce proteins passing through the Sec secretion system in biotechnology, although *Bacillus subtilis* does not have SecB as a Gram-positive bacterium and uses CsaA instead of SecB as a chaperone [70–75]. Some studies have been done to coexpress SecB and SecA from *Escherichia coli* in *Bacillus subtilis* to produce proteins in need [76, 77], and a similar case can also be found in *Salmonella typhimurium* [78].

It is not clear whether the solubility of SecB is a factor in the evolution, because SecB is a soluble chaperone and the macromolecular crowding could affect SecB [79]. It is yet to know whether the evolution of SecB is directly related to the evolution of SecA because the previous phylogenetic analysis on 86 microbial genomes showed that 59 organisms had SecA but 31 had a gene for encoding SecB [80]. A possible selective pressure should be directed to the trigger factors DnaK (HSP70) and GroEL (HSP60), which competed with SecB for the same pool of newly synthesized polypeptides [81–83]. This is because both trigger the factor DnaK, and SecB shared potential binding sites in nascent polypeptide substrate [84–86]. Indeed, it was showed that cotranslational substrate recognition by SecB was greatly suppressed in the presence of ribosome-bound trigger factor, but not by DnaK [82]. Still, it was suggested that the general evolution of chaperones would favor the sequences that coded both the functional native state and folding intermediate with high affinity as well [87]. Another possible factor, which affects the evolution of SecB, would be the Tat system, which transfers folded secreted proteins and plays a complementary role with SecB [88]. In addition, the newly discovered toxin-antitoxin-chaperone (TAC) system of *Mycobacterium tuberculosis* was found evolutionarily related to SecB [89]. This is an interesting topic to pursue in future.

As a matter of fact, the above analyses are largely related to the vertical gene transfer. Currently, the horizontal gene transfer draws more and more attention, because it takes the advantage to transfer genetic materials not only between sister clans but also between distant clans. Therefore, horizontal gene transfer was considered as a major force in the prokaryotic evolution [90]. Evidence suggested that the genera *Achromobacter*, *Brevundimonas*, *Ochrobactrum* and *Pseudoxanthomonas*, which were included in this study, were found to be subject to horizontal gene transfer [91]. Also, the horizontal gene transfer was observed between *Stenotrophomonas maltophilia* and *xanthomonad* [92], both of which were included in this study. Doubtless, the partition of variance of p-distance into inter- and intra-clan variances could be the first step to numerically estimate horizontal and vertical gene transfers, because it was indicated that phylogenetic analysis performed poorly in the prediction of horizontal transferred gene in *Methylobacterium* [93], which was also included in this study.

**Fig 5:**
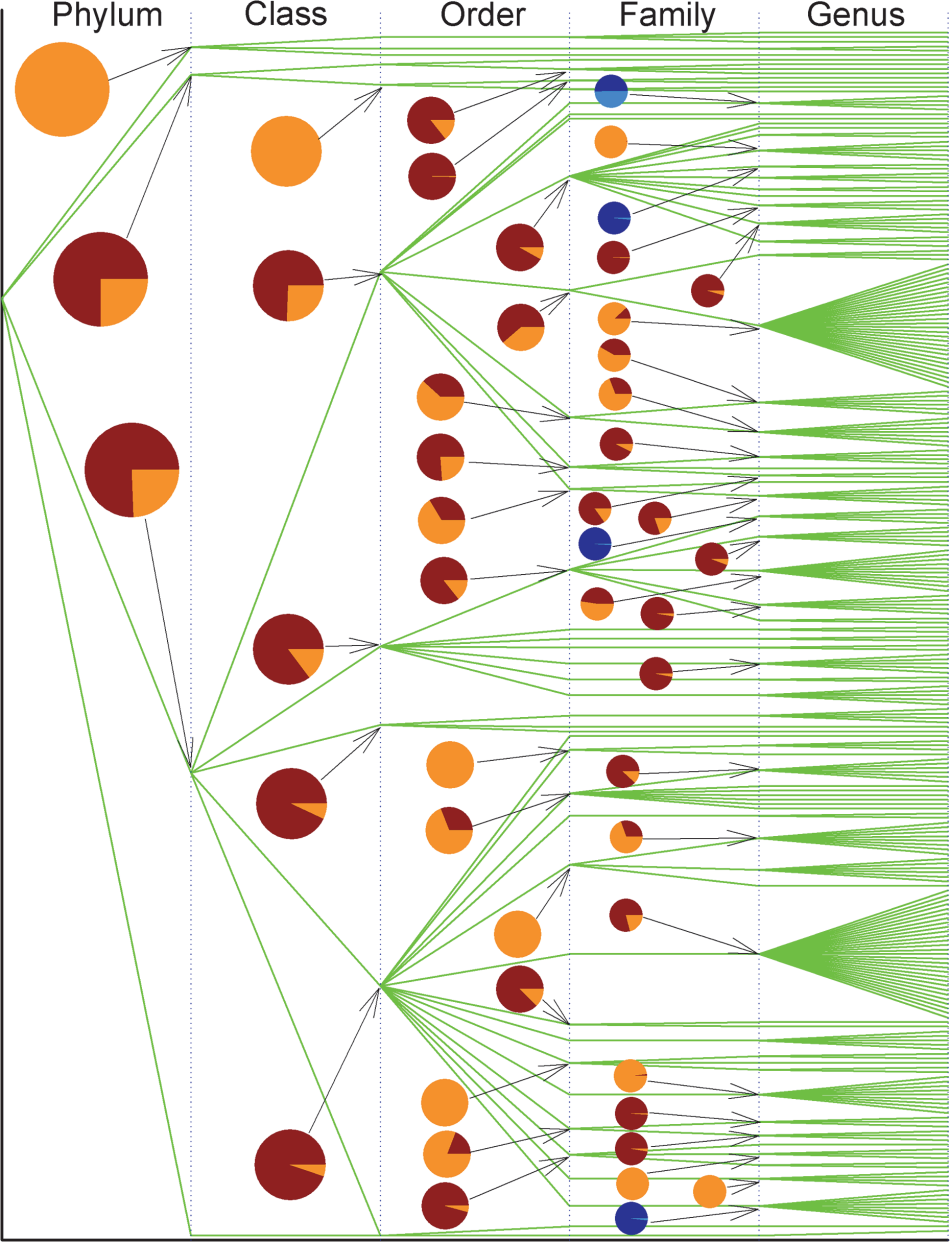
Partitioning of variance of p-distance into inter- and intra-clan variance along taxonomic lineage of bacterial SecB. The bifurcation is the point across taxonomic boundary line. Pies show inter-clan variance (dark color) and intra-clan variance (bright color). Taxonomic names can be found in S2 Table. The pies with dark and light blue colors are inter- and intra-genus variances for families *Caulobacteraceae*, *Brucellaceae*, *Alcaligenaceae* and *Pseudoxanthomonas*, listed from top to bottom.

Fig. 5 illustrates the partition of variance of p-distance into inter- and intra-clan variances by means of the model II ANOVA, which analyzes whether a SecB is more likely to evolve within a taxonomic group or across a taxonomic boundary line. In other words, this analysis answers the question of whether the SecB evolution is likely to be constrained within a genus, a family, an order, a class and a phylum, or across one another. In general, this figure can be read as follows: the larger the bright color area is, the larger the intra-clan variance is and the smaller the inter-clan variance is. This is because the sum of inter- and intra-clan variances is 100%. Accordingly, the larger is the intra-clan variance, the larger is the tendency that the evolution goes within a taxonomic group, whereas the larger is the inter-clan variance, the larger is the tendency that the evolution is more likely to go across a taxonomic boundary line.

Although there were 3813 bacterial SecBs used in this study, not every taxonomic group can be computed with the model II ANOVA, so not each taxonomic group was marked with pie in Fig. 5. For example, the computation was impossible for *Stenotrophomonas maltophilia* [92] and *Methylobacterium* [93]. In Fig. 5, there were 9 taxonomic groups whose inter-clan variance was almost zero indicated by the pies without dark color area, including the phylum *Bacteroidetes*, the class *Clostridia*, the orders *Aeromonadales*, *Chromatiales* and *Oceanospirillales*, and the families *Bradyrhizobiaceae*, *Pasteurellaceae*, *Piscirickettsiaceae* and *Vibrionaceae*. So a SecB in these taxonomic groups was more likely to evolve along the line within their own taxonomic group. On the other hand, there were 11 taxonomic groups whose intra-clan variance was almost zero indicated by the pies without bright color area, including the orders *Clostridiales* and *Thiotrichales*, and the families *Brucellaceae*, *Phyllobacteriaceae*, *Rhizobiaceae*, *Alcaligenaceae*, *Oxalobacteraceae*, *Neisseriaceae*, *Moraxellaceae*, *Pseudomonadaceae* and *Xanthomonadaceae*. So a SecB in these taxonomic groups was more likely to evolve across a taxonomic boundary line. For the rest of the pies, the cut-off line of 50% could be set up, suggesting the evolutionary tendency that a SecB could evolve within a taxonomic group or across a taxonomic boundary line.

For example, there were four genera *Achromobacter*, *Brevundimonas*, *Ochrobactrum* and *Pseudoxanthomonas* belonging to the families *Alcaligenaceae*, *Caulobacteraceae*, *Brucellaceae* and *Xanthomonadaceae*, respectively. Previous study demonstrated that they were subject to horizontal gene transfer [91]. The current study showed that their inter- and intra-genus variances were 98.99% and 1.01%, 50.35% and 49.65%, 98.45% and 1.55%, 98.22% and 1.78%, respectively (Fig. 5). These data were further evidence for horizontal gene transfer in these genera, and those mobile genetic elements might lead to mosaic-like genes. Thus, statistical analysis provides very interesting results that not only provide additional proof to support the conclusion obtained from phylogenetic analysis, but also throw new light on other issues.

## Materials and Methods

### Data

A total of 5162 protein translocase subunit SecB sequences were downloaded from the UniProtKB, and this amount was all available SecB sequences in the UniProtKB for the release 2014_01—February 8, 2014. Among them, there were 5141 SecB sequences without the annotation of fragment, whose average length was 157±27 (mean±SD), and 21 SecB sequences with the annotation of fragment, whose average length was 113±50, so the latter ones were excluded from this study.

In order to accurately, precisely and reliably explore the evolutionary relationship of SecB sequences, only the ones that had full taxonomic classification from superkingdom to species were included in the phylogenetic and statistical analyses in this study. For this reason, the taxonomic lineage of the remaining 5141 SecB sequences was verified against the UniProtKB for release 2014_03. This verification revealed that one, three and 3813 SecB sequences came from archaea, eukaryota and bacteria, respectively (S1 Table). They had full taxonomic lineage from kingdom to phylum, to class, to order, to family, to genus, and finally to organism. These 3813 bacterial SecB sequences covered 4 phyla, 11 classes, 41 orders, 82 families, 269 genera, and 3744 organisms (S2 Table).

### Phylogenetic Analysis

The alignment of 3813 bacterial SecB sequences was conducted by using Blast, Mega [94] and ClustalX [95], appropriately. The phylogenetic tree was constructed by using ClustalX with neighbor-joining method, presented using NJPlot [96], and validated by using ClustalX with 1000 bootstrap replicates. PyMOL was used to analyze 3-dimensional structure of two aligned SecB sequences.

### Statistical Analysis

The amount of 3813 bacterial SecBs can give very precisely and accurately statistical estimates on this large-scale population of SecB sequences. Accordingly, the average pairwise p-distance was computed using Mega software for each kingdom, phylum, class, order, family and genus. And then the model II ANOVA was used to analyze the variances of p-distances in terms of inter-phyla, classes, orders, families, genera, and intra-phylum, class, order, family, genus, because the model II ANOVA is particularly suited for this type of study [97–104], and SigmaStat was used to perform the model II ANOVA [105].

## Supporting Information

S1 Table SecB protein sequences with full taxonomic lineage used in this study. (DOC)

S2 Table taxonomic ranges that the 3813 SecB protein sequences cover in this study. (DOC)
