# Supplementary material for: Large-Scale Evolutionary Analyses on SecB Subunits of Bacterial Sec System
Source: PLoS One. 2015 Mar 16;10(3):e0120417. doi: 10.1371/journal.pone.0120417 (PMC4361572; doi:10.1371/journal.pone.0120417)
Supplement: S1 Table — (DOC) [file pone.0120417.s001.doc]

Table S1. SecB protein sequences with full taxonomic lineage used in this study

| R5U3B2, F3PW75, E4T2T9, J4KE05, I3ZA85, G0IZN5, H6L2M6, J0XWL3, J1I4F1, E3E5I9, U1DPB6, U1E7J6, I8R797, U2GJY0, D7V892, H3P0T0, K4PQI2, I0SWJ4, S7XBN7, H7IPG9, H7GY06, H7GRX5, H7IK67, H7HMU2, U5WE05, H7P0P8, H7IVW2, H7J1R2, H7J862, H7HA89, H7JKY7, H7P8J7, H7JSL4, H7JYZ6, H7PJG5, H7K512, H7NW75, H7KD22, H7KJF6, H7PDD6, H7H4F7, H7I6I8, H7KMA1, H7HTT1, H7PQZ3, H7PY21, J1HCB4, H7QN05, H7Q3Z5, H7L257, H7IDY3, H7QB37, H7HGJ5, H7I0F8, H7LDT0, H7LM17, H7GKM9, H7LQJ3, H7LXV6, H7M433, H7MAX8, H7MFC4, H7MP19, H7N0H1, H7N5Q9, H7QGA2, H7NAE9, H7NI84, J1S9N6, H7NPW7, J1GD94, J1RW80, J1H1H2, J1RY53, I0SXX1, I0T351, I0W387, I0W5B1, R4K0U1, L7ELF2, D4LTG0, D4LUG1, Q2RKP2, L0III5, V4Q1D2, F4QJQ6, V4Q9K0, V4PY84, V4PJN0, V4QP06, U3AMH5, L1QQZ5, F4QZU5, B4W5N4, D9QJR9, Q9A224, B8GW20, R0EAG3, D5VQ80, B0T6F4, B4RBG0, A0LD65, E0TF68, Q1YE54, J0PPW2, M1NYG0, A1UU92, J0Q0U6, N6VHT3, N6UP89, E6YG07, J1JG69, Q6G540, J0ZT23, Q6G0W9, K4HHS8, E6YXN3, N6UR71, E6YP33, J0YYY2, J0ZRA2, J1K696, J0REP9, J1JXV3, M1N5R6, N6VQM0, J0QPX0, B2ICZ5, B8EQM2, K8P4V8, K8NIJ6, Q89WN7, M4Z073, A5E8G0, A4YJU2, U1H6H2, U1HDZ9, U1HNY0, U1I8F7, U1IFJ9, I0FY34, H0T985, H0TJV3, I2QKE3, H5YCQ3, J3I3I2, Q1QRY0, Q3SWG7, B6JAL0, Q6ND07, Q07UP9, Q21CL3, Q13E29, E6VI70, Q2J350, B3Q8B5, P0C126, B2S971, S3VR60, S3W457, S3PZB4, U7VKZ1, U7VI25, U7ZCV4, U7YC12, N7RG97, N7TBU0, N7TCV1, N7SDE9, N7TVE6, N7T9F3, N6ZCK6, N7V0Q0, N7ABR3, N7U2T1, N7UB56, N7UI99, N7B561, N7VZC5, S3SPP3, S3SR93, N7UX48, N7WHY5, N7B4E9, N7WT28, N7BJR3, N7AW54, N7X1G4, S3SKY0, S3STE6, N7C183, N7XCC6, S3WS51, N7CBX0, S3WBS5, S3W8D0, N7BEI0, N7X7F9, N7BVB1, U8AC65, N7D4T3, S3QS91, S3RKV3, S3S695, U7WLI0, S3R153, U7YQJ7, U7XAF4, G8T1J6, S3P1C8, S3NZT3, U7I596, P0C125, H3Q7K0, H3QGP7, H3QUZ4, H3R5U0, H3PBB3, H3PXB5, C9UUX9, C9UPZ1, C9UFP9, D7H0H3, C9VWN4, N7DGR8, N7CMG6, N7DWM1, N7F1S7, N7ECN6, N7X7G2, N7Z0M6, N7FG84, N7FTJ5, N7EXY6, N7G767, N7YHY0, N7YSE1, R8W963, N7FP68, D0AYL0, N7GY77, N7HEL8, N8A7C8, N7GQD2, N7HPB7, N8ALY7, N8ANQ8, N7I868, N7HQV6, N7HV16, N7J4Y3, N7JL26, N7K4T2, N7J7J7, N8A3B5, N8K8L2, U4VGK4, C4ITR0, A9M9Q7, U7YRA5, N7ZXI4, U7YA72, N7KPP3, N9TMZ4, N8BFH7, N9SMU0, G8SPJ8, N7JUK9, C9TDE9, D1FGS4, C0G320, E0DJG9, F2GU61, U8A3C4, U7W401, U7X9H8, N7KGX8, T0E170, N8BVL3, D0B3W1, Q8YE23, C0RFW0, D1F0L7, N7LU61, N7M302, N7L8A7, N8CB38, N7MN93, N8B1X8, N7NH43, N7NF87, N8CVV0, N7P0Z6, N8BS81, N7NY62, N8C150, N7NBA7, N8C6A4, N8DVY2, N8E2A4, N8D9D9, N8EM24, N8DTS2, A5VT30, N8FFK2, N7NJQ6, N8LZJ5, N8LBQ3, N8KNP0, N8MKL3, S3TAP8, N8M942, N8MV27, C9TWM2, C9TNP3, U7XRU7, N8EM59, N8EMM9, N8G5B0, N8F8W2, N8GNT0, N8H2I7, D6LQT0, N7PXM3, N8G263, N7Q9X9, B0CJH2, U7WJ65, U7WNM3, U7Z280, U7ZGA8, N7QFP7, N8IAU9, N7R019, N7QYQ9, N7Q4D0, U7ZJM4, Q8FY19, D0BE83, D0P9R8, N8IVW6, N7RY56, N8HXQ4, N8JEN3, N8IHZ2, N8KGP5, N8IWM9, N8KV62, G8NJQ0, A6WX66, U4VA61, C4WKL0, M5JTE9, M3JTI6, N0BHI6, G4R7R3, E3I7H2, C5AZH8, B7KV30, C7CAT4, H1KKR4, M7XPX8, B8IHW8, B1ZHA5, B1LWY2, B0UJF1, I9X0H2, I4YZ11, J7QU18, Q11CM1, G6YHU0, L0KE30, E8TGU3, M5EIR5, F7YA18, M5F257, Q98DU8, I5BWP4, K2N439, K2QJ02, B9JG28, F0L2W7, F5J6S4, Q8UJC2, H0HCZ4, G6XU30, M8B534, M7Y6M2, U6B4F6, M4QDR2, L0EXH4, Q2KE96, B3PVT7, S5SD89, N6U1Q6, S3I9W6, C6B102, B5ZV56, J0KKU2, J0VNL9, J0H4E3, Q1MNF1, J0V6E7, K0Q1D4, L0NKE2, J2WDB8, J2I8D4, U4PZK1, H4FA93, K0W0T2, L0LH49, V5D020, Q92TE7, C3MB76, I3XES0, A6UEF8, F6E5U2, F6BPK8, F7X2E8, H0G3W6, A8I1W4, D6ZYW8, A7IGA7, Q0C6A3, Q0AKD3, A3UIB8, K2JW57, I1B1J6, D0DA70, A8LPB5, Q28W02, F9Y694, E3F149, A0P3N0, U7FPS0, U7HEQ9, U2YM65, A3VD54, A9DYT8, Q2CJU9, K2HB44, M9RNU9, S5YG80, A1B5S4, Q0FVB0, I7E5N9, G8PM48, B6R215, D5AKF2, L1KCP5, C8S213, Q3IYG6, A4WVZ7, A3PNT4, B9KPW0, J6LJY9, E2CHC0, Q16CY7, A6FL46, A4EGV8, B7RKV7, A3X7D2, A4EYL7, A3SKU7, A3W999, A6E522, S9SDU9, R0F3F7, Q5LLN4, Q1GCM7, B7QU47, D0CSK7, C9CY80, A3K2Y5, S9QSN3, A3SUI3, S9QCH7, C7DEX2, C7K631, C7JEA6, C7JEA8, C7JEW7, S6D4E6, C7KV95, C7KV97, C7KYY1, C7JLM1, C7JQG6, C7JVT9, C7JZN1, C7L523, C7L8Q5, C7K2E9, C7KBL8, C7KKY4, C7KPL6, H1UJX6, H1ULT4, H1UPC9, F1YSF0, F1YT66, R5XSR6, R5QJ87, F7VGY5, F7VIB8, A5FZF9, F0IZH2, F7S3Y5, A9HK92, D5QCZ1, F3SBB0, G6XGH8, G6XIU1, Q5FNS6, Q5FPS3, K7SLT3, K7T5J9, M9MHU1, T1CZK7, Q0BPL8, D5RLD4, G7Z6Z3, D3NYP8, R6I162, K9HVF7, A4U2B6, Q2VYH7, K2J5Y7, S9SGF2, B6IU92, H6SMK7, Q2RN91, G2TEV6, K2LYT0, D1AT14, B9KHK9, Q5PBR8, U5XTD8, U5XSB8, Q2GLM6, S6GBN3, S6G505, S5PXX2, S5PIW4, Q3YR47, Q2GHM9, Q40K61, Q5FG94, Q5HAD9, C6V4L7, Q2GE48, B6Y729, M9WYJ7, M9WXQ4, C0F9S0, I7IU35, B3CMS4, H0U3S1, Q73IQ0, U6SWQ3, Q5GS95, C0R5F0, F7XVH8, A5CFN0, B3CUK7, C3PMB1, A8GM17, H8K441, A8GUI7, Q1RGY7, A8EXD7, A8EXD8, H6PDK2, Q92JG7, C4YYB8, Q4UNF2, G0GWN5, G4KLZ1, A8F0H7, U6EGP4, H8KBH7, H8KEF3, C4K189, H6PVY1, Q9ZE76, D5AVZ3, R0M612, H8N2I9, H8N9V5, R0KIZ2, H8ND58, H8N652, M9T707, H8NGE5, H8KHD4, B0BW24, A8GQN4, H6PR81, H6PJT7, H6PLH7, H6PX58, H6Q1V7, G8L9B1, H8LP48, Q68XU4, H8NKP3, Q2N7Z9, A3WFI9, Q2GAU0, T0J6F2, F1Z647, G6EAC0, J3ANK4, F6IIM4, I9WGG8, T0HW78, F6ESQ0, D4YZ81, N1MFU4, T0ITS5, T0H721, T0GQ03, T0JB54, J8VP99, M4S418, F3WW03, Q1N8S1, A5VD08, Q1GRJ6, N9W408, Q9EZ97, C8WEH9, I6YWJ5, H0FCZ8, D4X4F7, J4PAS2, E3HLN1, E5UGP5, R4XPN5, M5IUF0, Q2L0A9, Q7WQN5, K4QGG8, K4T8T0, M5P0Z3, Q7W1Q9, J7QH16, Q7VS46, A9IFJ5, F4GQG5, I7IC63, I7IJI8, I6XC46, Q0BBK8, B1YNA3, B1FFS4, B4EA67, Q1BTB4, A0KAS7, B1JZ58, U1XQY4, G7HTN5, A2VV67, J7IZ22, C5ADP9, Q62F46, A2S628, A3MQ26, A1UZX6, A5TIM1, A9JZY2, A5J6D4, C4AYU4, A5XSB6, C5NG22, A9AEK0, J4JK56, J4SA59, B9B3F3, B9BVM2, B2JC98, B2SX18, A3NR12, Q3JWH4, A3N5B3, Q63XU4, I2KLZ2, I1WN00, C5ZJ21, I2L4U4, I2L9D5, B2H1P7, A4LJQ1, I2MJZ5, I2M6T7, A8E919, K7PSP7, M7EMS1, S5NYN1, C4KPC4, U5UVW8, C0XXT6, B1HG94, E5AME4, Q39CN9, D5WAR7, E1T5Z8, E8YLE0, I2IDA4, B5WP66, F0GKN1, G8MAS6, Q2T1H2, I6AKU9, A4JI42, Q146B7, G4M980, Q0KET5, Q476J4, L2EMG8, V2KID5, B2AGQ0, Q1LRT6, E7S0C5, A6GLR7, G2ZN85, C6BBC7, B2UE35, U3QCR5, Q8Y2I0, D8NQ45, D8N8R3, B5SDY5, V5AG95, A3RSA8, U3GHX1, E2SW43, I9WA81, F0Q0Y7, A1TTW2, B9MEY9, A1WBJ0, J1E9Z9, K0I4T6, H0BTL0, Q21YV7, E8U1A1, D0J5Y0, B7WVG5, A9BUZ6, S2XAB7, S2WFJ1, F6ARQ8, F3KPK0, A1VKR9, A1VKS9, Q12F47, J2TZY4, F5XWP0, E6UYM5, C5CWV6, A1WDX7, D8IUW7, J2UTP3, I3CMQ7, J3HXC7, A4G995, A6T312, L9PK24, K9DGU9, C3X5K3, C3XBS7, F3QHD6, H3KHD8, R7IJA3, R7C3P3, R7KKX4, K1JJB2, E7H0W1, R5Q5P2, S3CA84, D9SIZ1, D5CNE0, S6AB29, Q3SG95, Q1GZ85, C6WU78, D7DN18, Q7NYZ5, F0EZZ4, F5S9U8, H8DZ36, C4GLV4, C1DDI6, F2BAR8, D0W2V2, C0EQW5, C5TKF2, Q5FAB1, B4RQ47, C1HVM5, D1D3S3, D6H9J2, D1DAW0, D1DU55, D1DMB8, D1E0M9, D1EDE9, E4ZEZ1, E1P0X5, F9EUS3, C6S5D0, L5SMX4, R0T669, R0XQ56, R0WUG7, S0GAZ5, R0YBF6, R0RN77, R0U5V0, T0VWM4, L5QKL7, R0SIT3, R0XD06, L5TFD0, R0VUN3, R0WDP2, R0W1B0, R0Y871, L5QFX4, R0WSZ7, L5SPC6, L5TM91, R0QYN3, R0R6R0, L5QGW8, R0RH38, R0Q244, L5PW09, L5TRA3, R0QPD7, R0PCH6, J8WZM6, R0N0D3, L5V1N9, R0Q0U8, R0V6D1, R0VFJ8, L5URZ3, J8XDY6, L5P437, L5PMW2, J8XXZ9, J8WA06, J8VWI0, L5S6Q8, L5T621, R0Q4H3, F0B0K4, R0QMK8, L5QX83, R0RA06, L5TYD9, L5PMT8, J8U2T2, C6SC29, C6SMW0, I4E7Q9, E0NAW3, F0API4, F0AIR5, R0UFW1, F0A777, L5RK27, L5RXJ7, E9ZW83, T0XST7, T0WN80, R0Z7B8, R0S2V7, S3MA28, J8WRI4, R0V2B0, R0V8F2, T0W3B0, R1ARG2, L5RNR1, J8TD71, R0ZKQ3, I2HDT3, J8T5E5, J8XLB6, R1ANG4, R0YRX0, J8WTX7, J8YG84, T0XAH1, R0XT90, J8V1S9, R0T733, R0ZPA3, T0XF66, T0VG60, R0ZSJ2, R1A721, R0ZYY7, T0YMD7, R0ZMH3, R0Z9E4, L5UIZ4, L5UCZ6, L5R1T4, L5P3K1, R0TVZ7, R0W4C6, T0YA17, J8X209, L5R6N4, R0T1B0, R0S547, L5RAS7, R1AWU1, R0UD95, R0Z5A4, R0UKV3, F0A1U8, E7BEH7, Q9JVU8, E3D2A8, F0MF20, F0MPD8, F0N002, Q9JY16, F0NA10, E6MYD9, A9M1W7, C9X1B8, A1KSA7, D2ZTC8, E5UHI1, E2PI26, G4CK09, I2NWQ0, G3Z4M3, L1NVT2, G4CN63, B9Z6D1, G2IYN4, Q82SU4, Q0AIA2, Q2Y9Z4, Q5P7N1, A1K9C3, Q47IG1, F5RF48, N6Y8C5, N6Y847, N6ZKC3, C4ZPA3, N6XHL4, N6ZDN8, N6X3R0, T0ASP1, E8RFW3, M1NC67, Q6AS31, D6Z6Y4, S7TD18, B3E290, A5G9X8, F9ZMJ7, G0JPU7, B7J4Y5, B5EKH2, F8XRR4, R4VIS9, K1J437, A0KF07, K2I490, R1H9J3, A4ST21, T0QT58, G7CZI1, F4DGE2, K1IQI8, K1HV00, K1J0W8, U1H278, H2G055, C4LA08, E8LKB3, R5EIY7, R9PQS3, J1YEG8, H3ZCV1, K0CX69, B4S0I7, K0CN04, J9Y7P1, S5AAW0, S5AYX6, S5BHS8, S5CAM2, K6WUL8, K6XHX5, K6XR59, G4QJJ0, K6YY09, H5TEY1, F4ATK4, A6EZE4, A1U5G9, H8WEY2, G6YMS2, U7P5S0, A3JBM4, U7GWD0, U7G387, U7NRN9, Q21NG8, Q47VY5, E1SR17, A3WJ12, Q5QZA7, K2K4C6, U1J831, Q15PS2, U1JN08, Q3IIE1, F3BDW6, V4H5L5, U1L286, U1KH06, E6RJ14, G7F396, G7FP38, G7EMR3, M5H0V3, U1M2W1, U1LCX4, A1SZI0, A1S1L8, A3DAM8, A6WHC9, A9KUC0, B8E3F6, E6SYK1, G0APS6, H1YUS8, G6DWU6, A9D4S7, Q12HV7, Q07VX0, B0TLE8, A3QJM2, Q8EKP0, A8HAD8, B8CGT2, E6XK79, A4Y1E5, A8G1U7, A0KR79, Q0HP89, Q0I0Q4, A1RQ93, F7RJH0, B1KQ11, C8N8B4, G9ZCH8, A5EW94, D3RRL7, D5C459, Q3JF20, B6C663, D8K828, I1DT49, F7NYT8, F9UCB7, I3YCI9, L0H403, G2E1K5, H8YZF8, H8Z4S9, Q0A5H6, H1G0G8, A1WWC6, R4V4V3, L0E2D0, B8GR95, D3SC16, G4E606, D0KX36, D2U335, G7LU02, G2LMD3, G2LNU2, B8D8L1, P57161, E3JJH7, E3JH59, E3JDR5, E3JFP5, B8D6W5, P32002, Q491X9, C4K4M5, E0WSW1, G2GY32, S3IYE8, G9SBY5, K8QZV2, M3DTI5, R1FPB1, A8ARJ6, D2TIZ3, C1MDR7, J1G3L1, R8WJU8, R8UJV4, D4BIN7, K8ATQ4, K8BPA6, K8AAY2, A7MID6, K8D358, K8D1T7, F5VRC6, M1J123, C9Y376, K8DT67, C6C6S8, U6ZPC5, C6CNE7, C5BC34, D0ZGW8, E0T253, D4FAS2, M4TNE4, M0Q8X6, G0E7W6, L8BNY0, G2S8X5, D6DP82, V3J4N1, V3JFP3, G8LFT5, S7V255, J7GF22, I4ZG46, V3DVZ3, V3DQM3, V3H6M8, V3IH14, V3EZ15, A4W536, J0MB98, U7CUR9, V3RY49, V3REB0, V3R113, V3PRX7, V3PNP8, V3PCM3, V3LWP4, V3MF55, U7CKH6, D4IBT1, N0F634, E5B0B1, N0FEN0, N0EVG4, N0EH76, N0E7T5, N0G1F4, N0GHF1, D8MKG1, D2TC19, B2VL52, R9NU68, B1EHX5, Q8VVM0, B7L735, B1IZI1, E0J5F2, C6EDI0, C6UHC7, G7RQ82, P0AG86, B1X943, C4ZXK1, B6I3I8, B1LK48, E1RU86, Q1R4Y5, K3JBX6, U0MF97, U1B7D5, U0GFF7, K5K3G8, K5HIC9, K5J7P3, B3X8U2, U9YX47, U9YY52, V0RI67, U9Y115, I2SUE9, M9EFD2, N2D8Z0, N3I940, N2LKU1, N1SIA9, M9CLM6, I2X6X5, I2Y5B9, G1ZBG6, M9K747, M9KNX1, N2ENX8, N1T8I8, N2KGH5, N2MX31, N2MPU6, M9CD23, N2MM26, M9D3A8, M9BR53, M9B527, M9AVG9, M9AWF5, M8Z5I2, M8YBT0, M8Y0L1, M8X7Y1, M8WNM9, M8VWB5, M8WDB9, N3IW81, N2P315, N2NWW7, N2Q543, M8TBX3, M8TDD9, M8T787, M8SH74, M8RVR6, M8QRP6, N2QGG2, U0RJ45, K3IB24, I2YFX1, I2TI50, I2XSU8, E6B1C1, L9HV94, H1C393, I2UC07, B2N751, I4TPM3, I4SJ73, K3HSG0, I4U4H3, K5FAQ4, K5J9T4, K5FF91, K5FH52, K5GMJ4, K5IW05, L0X9Y1, L0Z3Z4, L0YKZ8, I2WAS8, U9YQG1, V0S687, V0S2E9, V0SQE6, V0S7M7, V0T9Z5, V0TZ58, V0AMV4, V0UAI1, U9ZW45, V0UQ66, V0VCK0, V0VCJ3, V0WNU2, V0W5M4, V0XDG7, V0XHY4, V0YDE5, V0XA76, V0Y8X6, V0ZQX4, V1ADD5, V1AI91, V1B6M0, V1AT25, V1BLN3, V1BPE5, V0A1S8, V0AVL3, V1BM30, I5ET14, L1BGF1, L1BRG7, L1R7N5, L1CXK4, I2T221, L1DWS2, L1D9X6, I2VKN4, L1ELS8, L1FE79, L9ILX2, L1FZS9, L1GP68, L1GSM6, L8YHK6, L8ZAP9, L9B1I6, L9GQA6, L9BFI0, L9CQ15, V1D638, V1D6T4, K5CJG0, K3J4T2, R9EKK3, U5B2V9, L9CJ35, V4UV03, D6I2M6, H1DTG5, U0GS62, U0RL63, U0FG14, U0TYI6, U0T4L3, U0VJU1, B3WKZ4, D6IFJ5, U0HAL1, U0HSI2, U0H2R4, U0IBH1, D6JGS5, U0JS43, U0J287, U0VR58, U0VM27, I2ZM22, U0VZB8, U0JQB6, U0K8Q4, I2PGJ6, B3HGL6, U0YE85, U0XKR1, U1B3H6, U1BAH7, U0M612, U0MSK2, M8QP86, N3KKB9, N2QHE8, N2HV98, N2R7C9, M8RBK6, N2RSB0, M8PSZ1, U0MPP6, U7CA36, V2ZE46, V2ZZH5, V2YPT7, U7BT79, V3HI33, U5M7F6, R6TT94, V5DX49, V5DF86, V5D543, J7RY88, I4UUB0, H5DS63, H5E9E5, H5ERQ5, H5F6M6, H5FMD1, H5HEW0, H5IAH2, H5J9C6, H5K674, H5KKT7, H5L0F4, H5LD25, H5LTG2, H5M7J8, H5MM90, H5N0M0, H5NG20, H5NVB0, H5PSE6, H5Q5V1, H4I2C4, H4IHU8, H4IYT2, H4JUF1, H4K975, H3KVG0, H4KNN9, H4L4I7, H4LIW3, H4M026, H4MGP4, H4MY23, H4PBT0, H4PR97, H4Q860, H4R5Z5, H4RM88, H4SZA2, H4TEL7, H4TVG7, H4U8M5, H4V7P7, H4VN17, H4W348, H4WH07, H4WXT9, H4XS89, H4YMX9, H4Z3D0, H4ZKU1, H5A3R4, H5BHR4, H5CD11, H5DA49, B3IIT4, E7HWQ6, E9WLT2, B3IC01, S4A2V6, E9WZZ9, I5YJZ7, K3L6M4, K3L711, K3LIP1, I5XZS7, I5ZE56, K3LUP7, K3NBH0, K3MT85, K3MZI0, K3P446, K3PR73, I5ZCQ7, K3PAV8, K3REU5, K3RC55, K3S860, K3SMQ5, I5VB44, I5SR75, I5VMA3, I5U7K3, I5UZ31, I5WNH6, I5XDF0, I5W8L3, I5XHZ8, K3GU75, M9K0B8, M9J3R3, I6GG45, I6FU44, E7HDX4, B3HMI0, K3A8B4, I5DFV5, K2Z530, K3A4X4, I5F1Y9, I5DD53, K3B6A4, K3B8L1, K3TKQ9, K2Y808, D7JVA3, G2BFE8, E9VEE0, F4W1G2, H1ERS2, E9Y5Q1, H1FBB2, F4VKS5, I2PG69, T5M1J1, T6PP80, T6QMZ7, T6RQE6, V4FS79, T6R819, T6S2M5, T6TH06, T6UTS9, T6US54, T6VE52, T6VM16, V4C9T4, T6WX53, T6XLS5, T6YIP9, T6YTT6, T5QH51, T7B671, T7B325, T7A5N2, V4C0Y4, T7BDR8, T7CES8, T7CH40, T7CK19, T7E9B0, T7ERG2, T7FMA8, T7EYE5, V4DV84, T7G0T0, V4DRP8, T7GD58, T9Y7G5, T9ZAD5, T7H543, T5QLX5, U0ACB5, T7IWD3, T7KP59, T5R7L3, T7KVD5, T7LVG1, T7LZX2, T7MQB2, V4D745, T5RYC5, T7P0J2, T7NHE1, T7PJE7, T7PI44, T7R0Z1, T7S5X5, T5SC02, T7RLF6, T7RP31, T7T877, T7TN37, T7TKA8, T7UA78, T7V9M8, T7V035, T7U7L0, T5RWV4, T7V620, T7VXY8, T7X4C2, T7YPN9, T7Y9E7, T8AG69, T5SI17, T9ZMG6, T7ZXU1, T8A517, T7ZZZ0, T8AR61, T8AZR2, T8BQE6, T8C637, T8E125, T8DT40, T8EGN3, T9ZWH0, T5TSD1, T5TVF5, T5UFF3, T5UFV5, T5VGF0, T5V4M8, T5VSQ7, T5WEQ8, T5WMS3, T5XM72, V4BL45, T5YKT4, T5N8R8, T5ZL76, T6B3A9, T6B6U8, T5NDI4, V2T5Y5, T6C9N3, T6CTF4, T5NGH3, T6EBE1, T6G2D3, T5PI56, T6H7E4, T6HD46, T6GRG4, T6HZ67, T6IGJ1, T6IEV2, T6JXP5, T6KGY3, V4BP07, T6LBS6, T6L358, T6L5W1, T5PBE4, T6M6R3, T6N2A1, T6MQ65, V2Q656, L5VQB7, U5SQV6, M9I746, I4ST79, I4SXU5, U0AMM4, T8LIJ3, U0BM34, T8EDF9, T8FJW9, T8FGD8, T8GZ41, T8G2I6, T8HNL0, T8H8L7, T8H3V0, T8IG99, T8IW31, U0AJT9, T8J3H6, T8J4J8, T8JW39, T8JT29, S1GFN1, L4FR63, S1I4R7, S1H2X5, L4TJ97, L4U0M0, S1IWH0, L4UK35, L2VNW4, L3TH14, L4V4Z1, L4FSP3, L3TJU7, L4VFJ2, L4G769, L3UB71, L4W5K4, L4WWU8, L4H2B3, L4WGK7, S1JP39, L4XHI1, L4XTE9, L4YID9, S1LQH3, L4HFU1, L4HCZ9, L4Z2T7, L4ZHT3, L4ZYL4, S0UVT6, L3UJY5, L4IJQ6, L5A8B3, L4IXS3, L4JFJ3, L5AUE5, L2WDL5, L5AXS7, L5BD16, L4K3I7, S1LJX9, L3V4Y3, L5C0B9, L4JUA5, L2WC70, L3WAG3, L5CB36, L4L4N9, L5D3K9, L5DAS0, L5DHU2, L3WLG5, L3Y795, L3WMJ7, S1MXM0, L4LG25, L5EG78, L2ZDT8, L5FG22, L5FHZ1, S1NX06, L4NHI9, S1RWM1, L3A149, L3AYE5, S0UYH3, L4LL62, L3BFV1, L3C0J6, L4L0F1, S0YGS0, L4MUL5, S0ZQN4, S1ABR7, L2TZJ2, L3C4X1, L4P361, L3D2Y4, L3DEQ6, L4PX63, L3DWB1, L4QGC5, L2X6N3, L3E138, L4QSD5, L3EQN8, L3F3A2, L4R595, L3FM98, L4QVG5, L4RQL4, L4B282, L3GBR9, S1AW30, S1BIJ0, L4RZP8, L3GK36, S1QNS6, L4SC57, L3H730, L4T576, L3H7I2, L3I4T2, L3HWH0, L3IPK2, L3JAV5, S0W3T8, S1QTH8, L2X275, L2XYN1, L2Y3U4, L4AN64, S0WS14, R8WRM7, S0X3J2, S0TGC0, S0X5N6, S0XP49, S0YAZ7, L2U3H8, S0Z6P8, S1NJ97, L3ZIJ6, L4BHM1, L3JLK8, L4BK20, L3K7K0, L4CX47, L3LES8, L3LYB1, L3M6G7, L3MC30, L4CXQ0, L3XS63, L3MXH4, S1CK55, L3N460, L4DQZ6, L3NVB3, L3PER0, S1DDE8, S1D3V5, S0STB9, S1E922, S1E8E9, S1F2I2, L3PVC8, L3Q841, L3QLV1, L4DXX7, L4EWL8, L3R9E9, L5GK25, L3RZU3, L3S2U7, L3SPG5, L5GW78, S1FU26, L3Y2N9, L5HRR5, L4F7G8, L5HZW0, L5IY02, S1GH55, L5J8D9, E7I7P7, F4T5G5, I4J5E0, K3EDS1, M9HDY2, M9I9Q2, M8PGR5, M8NUR9, M8QB98, M8MD19, M8N8V2, M8MC35, M8L019, M8LRU8, M8JU28, M8LDR4, M8KNJ2, N2SAZ7, D8EH11, E5ZZL2, D7Y7D9, D8ALG7, E1J6A8, E1IWX1, E6A979, D8AXX4, D8CAY9, D7ZNN0, D8C2S3, D8BHM5, D8ABU4, D7Z0W5, E9UAX1, E9TY44, D7Z9L6, E1HWD6, F8X687, D7XWU8, E6BP22, K3KG65, G0D6K2, E0R5E5, K3CKS2, K3SVP7, K3BAU5, L8CRS7, M2N5A5, A1AHE6, L8C9K7, C8TZA5, I4NIJ6, I4NEI6, U1I0T0, U1HRI7, J9ZH05, K0BUX7, F9CP16, G5U4B5, L1VUG1, L1W214, L1X3C7, L1XG13, L1YHS0, L1YLX9, L1ZIA4, G5VVQ2, G5WCB4, G5WKH7, G5XJ68, G5Y8Y9, F9HZ39, G5TP81, M7UTC0, L2APW9, L2BQK0, L2DCS3, F8YM86, C8UIZ9, M5HVK0, K4WJW5, I4NBR8, I4QWJ9, K4X1E6, I4QKW4, K4VRM4, B7ULG5, A7ZTG3, D2NCL0, E8HM45, E8I185, N6WEI9, P0AG88, B5YWB1, E2KN04, E2KCW0, B2PHK6, B2NKP9, B3AGH6, B3AX05, B3BRK0, B3BD21, E8J7R5, B6ZRQ1, B7NER6, D5D4B4, N1NCI0, M5I1B3, I4RPE4, K4WT01, K4UVR9, I4RIC5, B7MFH3, L8CJD4, H6MK14, E8IT67, P0AG87, Q0TBJ7, B7NPB8, G4PW23, B7M496, B7N255, A8A674, E1PJZ9, N2HEU7, N2SPB6, N2TYV1, N2UFU3, N3L736, N3KW70, N2UHP1, N2VPG1, N2VHW6, N2VMD8, N2WJW4, N2GLJ3, N2XCN3, N2XSA8, N2YM67, N2YPQ3, N2ZHP3, N3AD26, N3MA76, N3MGQ3, N2GKB0, N3AX96, N3BCI4, N3CB32, N3CBB0, N3DK00, N3DSK1, N3DDS6, M9FAM4, N3FB47, N3MX30, N2K1V1, N4NCD3, N2JQD5, N4NSG6, N4P7F0, N3F4Z4, N3N213, N3QN76, N1ST48, N3RR11, N3NT62, N3S200, N3ST05, N3FQS6, N4PWZ9, N4Q697, N3H1V5, N3GF20, N3HFF4, N3HVP3, M9DWL7, N3SGE8, N3TCK4, N3U529, N3UJ23, N3UPV6, N3V7Q7, N3VBB0, N3WSQ9, N3WYG9, N3Y9Q5, N3P618, N3YS14, N3ZQT9, N4B277, N4AXV4, N4QVT4, N4R773, N4RMD8, N4B756, N2FUV6, N4DFY1, N4DQJ3, N4ENQ5, N4ET82, N3PA90, N4FLA8, N4FTA2, N4G4E8, N4GIG5, N4GTI9, N4HKA7, N4I5H2, N2F299, N4HW54, N4IGT5, N4J9Q2, N4K8M2, N4KPS5, N4SAJ4, N4T6X8, N4SH70, N4LAT8, N4LMT9, H9UYG3, I5HFS7, L9CJR7, L9DP39, I5HUU1, L9DNC7, K3DMK4, I5JH47, I5JEI1, I5JW05, I5G2V9, I5KV10, I5KX51, I5L5T0, K2XJQ8, L9HJX1, K3IP96, I5M644, I5MSL8, I5MY89, L9EZG5, I5GCB9, K2YYK7, L9FMN1, I5GLX6, F7N2Q5, E7JKA3, M2NLD0, H8DES1, M2NSG2, G2A611, G1YFX1, G1YWJ9, G2AMD6, G2B189, G2BVV3, G2CAX4, J2XH03, G2CRM7, U6NEZ8, H0QE19, U0PC78, U0NQP7, U0QN38, E9YGB2, H1FPW3, F4UEW0, F4UUU4, F4V7V2, U0M9G4, I2ZG66, I5PHU1, I5QNR7, I5QZ64, I5SF73, I5RNZ7, I5PK52, I5Q225, I5TUV8, I5TQ81, K3HRR9, G2D5J5, U1DD06, T8KYL4, T8LZN7, T8MAE7, T8MD34, T8MB12, T8P1E4, T8PTA7, T8PZZ8, T8QYZ6, T8QMU1, T8R4U7, T8RN80, T8RS72, T8T0L2, T8SYK9, V4DNC4, U0CBB8, U0C020, T8STX6, T8ULZ8, T8U827, T8V6N4, T8V8T7, T8VJ90, T8XA74, T8XGL0, T8Z3G4, T9AVP1, T9BX18, T9ASS5, T9C022, T9DEC1, T9CLA0, T9E6S0, T9EJ64, T9EKS5, T9F8K5, T9GRG2, T9FLA2, T9HR05, T9G8T1, T9I2U3, U0CRQ3, V2SVL1, U0EB24, T9ICD7, T9J4Z7, V2T2X1, T9J265, V2RZU5, T9J8Y9, V2SKC8, T9JW51, T9KQH5, V2RGK7, T9L576, T9MQK3, T9MCH7, U1EYB1, T9NK01, T9PKD4, T9QDH0, T9QBC4, T9PXN9, V2TPJ9, T9R7G3, T9RER7, T9RL70, T9SN57, T9SHH4, T9SDV5, T9SWQ5, T9T216, T9WI21, T9V4N5, T9WC70, G0F2U6, F4M733, I0VWR3, G2F9E3, F9R148, B7LTL6, F0JW41, H5V415, U3G164, C1HSK6, G9Y959, H3LVD3, H3MCI1, H3MTV6, H3N4G1, I6WVY8, K6KJ88, V3QJH9, V3KVB9, R4YIC4, B5XTJ1, S7GU39, S7GAM2, S7FFQ8, S7IIN8, U6T729, S2BFG9, S2CQR1, S2C770, S2DBL8, M7PFV6, M7QAG2, U7BI37, U7AJH1, U7B175, V3CK60, V3C7U4, V3BTN8, V3B9T2, V3AZG3, V3BAY9, V2YR04, V2Z0V7, V3JY90, V3I9I9, U5MH77, S7A1V5, S7BAJ7, M2A888, M3ST34, G0GND1, U2BIA8, S1TS84, V3TBK9, V3UF89, V3REG5, V3QNC9, V3SGZ8, V3NUQ9, V3QAX5, V3NRB3, V3KZD9, V3LMI1, V3KGI3, V3J1A7, A6TFK4, G8VSF3, S3KZ98, J2F074, J1XRA5, J1YGX1, J2H0J6, J1ZYI0, J2BGR8, J2TXB2, J2JEE9, J2VCB7, J1VA77, J2N5E2, J1WWB5, J1WJI7, M5GIN9, S7YGU5, K4RVN1, K4SEC2, S7Y988, K1P395, K1PT18, C8SZX5, V3F2T5, V3D2W6, V3H7C1, V3GCN5, V3GIN8, V3FXS1, V3EWY9, S2G2H0, S7EUP4, S2G327, S7BUR8, S1TPS1, S7CTJ3, S7H635, S7DCF5, S1XGM3, R9BG35, S1VDH1, S1WH43, S1V2K6, S6YYL5, S7FK68, S2J9C4, S7AZH8, S1TIZ1, S2G3K9, S6YU43, S2ETU4, S7DTE4, S7AAP8, S7F191, S6ZU97, S7A4M8, S1XQA8, S1XU24, S1XUP2, S1ZKC3, S2A346, S1Z4P5, S1ZS80, S2A299, D6GL04, R8WQW5, D3RC33, J1YX86, L7BUB5, U4W2C2, F2EXM0, D4GE04, U4W9P5, G9AUI0, E6WAH7, E0M406, J3DK38, H8DJC4, J2UUB4, H3RIE8, Q6DAT1, C6DIA3, J7KSU7, K4FYZ0, D0KD39, J8T358, C7BSD6, Q7MY53, U7R5W9, T0QG72, R8ALT8, B4F139, S5U4S1, K1H8P9, K1GQ30, K8WR79, B6XJU4, K8W0R2, K8WGU5, D4C597, D1P7I7, K8WYJ6, I0DUL2, B2PVA6, H2IXJ9, B5EXB6, A9MKR5, F8VDV8, Q57ID2, B5FLH9, V2IW52, U6QFQ0, S5IS93, U6Q416, V2PEZ5, V2HK12, N1FJR6, V0KYH0, N1FDE3, V0KK02, N1EIX0, N1DZ34, N1D8B2, N1CUN4, N1CIQ8, N1FYR5, N1C5P6, N1BQ65, N1BBQ3, N1AQ04, N1IMF9, N1A5C6, N0ZU03, N0ZKP2, N0Z8V9, N0YS59, N0YA67, N0XYN4, V4A3N1, V3YHU4, N0XLJ8, V0GH59, N1I5M2, N0XAU5, N0WXL7, V0BMB2, N0VWX2, V0BVU0, N0V593, N0UVE1, N0UK82, N0U183, N0TJ42, V3XTX1, V3WWL3, N1HIR8, V3YRD1, V3WZY1, V3XFQ2, V3VGH4, V3W2Q2, V0DCL7, N0SHX0, V2G5Y9, N0T3N6, N0S8R5, V0F440, N0RWQ6, V0E169, V0DTS6, V0CY50, V0CJ82, N0RP58, V0CX05, V0BZY2, V0FFI6, N0QYJ4, N1ISK4, N0QBX9, N1H1F6, N0Q6R2, N0PGH1, N0P9R8, N0NQY7, N0NCN4, V0EIY2, N0MVQ5, N1GWQ4, N0M942, N0M124, N0LN25, N0LFW3, V0EWT3, N0KSG5, V2H0P5, V2FU00, N0K3U2, N0JL13, N0JKU4, N0IPD2, N0I6U5, N0HPG1, V2GYV4, V0I0J3, V0KYD1, V0M0J2, V0N665, V0IBP9, V0H6C0, L7B809, L7AWG9, L7AIU4, G5LVE4, V2GT12, V2HRR4, V2HJM3, G9WBS3, V2F7Z9, V2CQL5, V2F6R3, V2EH00, V2DM09, V2EDQ2, V2CAS3, V2NTC6, V2LFG4, V2BFZ8, V2AKD2, V2EI47, V2BZN9, E8NJD7, V2LLL1, V2BV22, S4IYX5, L9R5Z9, F2FCS5, M7RKU1, V2JDQ6, S4JAW0, U1T119, L9SRC3, L9S175, S4J7W8, S4J8J8, T2PR51, S4KP61, S4L7D9, S4LSW2, T2QJV7, L6QT57, L5X775, L6XJJ0, L9TMA2, L9TRJ1, J1RGY8, L6Z8E9, L6ZTF0, L6Q9T2, L6X4N7, L6RMZ8, L6W0T7, L6I805, J1PRE0, J2CPL9, J1JIU3, L6K562, L6ZP73, J1GSX8, J1J576, L6IRA6, L6PFS6, J1QG01, J1KF73, J2ELS6, L6VZ72, L6SUG2, L6TMU4, L6TUL4, L6XBW6, L6UUD3, J1X665, L6UIP9, J1W1C3, L6VG43, L6YMY5, J1J6D6, J1M4L7, L6YWN5, J2G456, L6BJM9, L6FTJ6, L6DEF3, L6ASL9, L6AZC0, L5ZTT2, L6DBV1, L6G202, L5Z4A1, L6A5F3, L6F5B4, L6GTX4, L5Z222, L6H608, L6BGM3, L6HMG7, L6D4Z0, L6HNK3, L6I894, L5ZUC9, L6GDQ9, L5XQC3, L6M1X8, L6NXW0, L6MBR4, L6L9R8, L9TED1, L6XXN6, L6L278, L5WRX1, L6N132, L9QBF2, F2FU13, T1YNV2, G5M9Z4, V2CEJ5, V2AFF5, G5MPY1, V2A985, V2NT74, V2L2T9, V1E772, I0P4F2, I0NEN3, I0LXN9, S5GQR9, V1E0V9, I0AEU0, K4ZYZ1, K5B3H0, K4ZQK1, K5B147, V1EJK9, G5N385, V1YQC9, G4C813, V1ZN42, G5NJS0, V1XYZ7, M4LQK7, B5NCV8, V1YJ14, V0J068, U6UN69, U6VJK2, U6U8W4, U6TL17, U6R3M2, V1YW95, V1X9A6, B3YDM0, U6URT9, V2NNP1, V2MR71, R7RGP4, V2MB31, V1VZC6, V1TJ58, V1UN53, G5PFA1, V1UH49, G5PUG1, E7YIQ5, E8E671, G9UR15, E7VGC5, E7ZX82, E8ACW8, E7ZIX1, G9V491, E8AKA1, E7VNH7, E7VW08, E7W5H3, G9VQ94, E7WI81, E7X1S6, E8BDL3, E8CIC5, E8B5E1, E8BVP1, E7Z3E5, V1UF28, G9TM26, E7YFV9, H0LHY0, H0M7B1, H0MMK6, H0N2C0, E8FKG8, E8FTE8, E8G523, E8GMH3, H1RA99, G9TTC1, E8CSI5, E8D5W7, E8DHP7, G5Q9Y7, G9UGH7, G9TCC2, V1JAI2, V1WE34, V1UTE7, U1IN18, V1WF41, V1WE23, V0FQ33, V2JS49, V2J284, U6Y0V4, U6X1L1, U6X1Z1, V0HTT1, I9ZHX5, J0G213, J0GAF1, J0CMA5, I9K595, I9Z3K0, I9RTI1, I9L5D7, I9HBR1, I9XYN5, I9E898, I9XZL1, J0ANX0, J0ESB8, J0GBB0, M3KWY4, M3L513, K0QG14, K0QGN7, V0NKF1, V0MZH1, V0MXZ2, V0MA27, M3L1R0, B4A570, S5SIB1, V3ZDU7, V0PSW9, V0Q8V4, V0PS42, V0NUP5, V0PBE5, V0N676, V2NKP7, V2LQV5, V1U102, V1SRN6, V4GKP7, V1RNH6, V1R3L4, S3DYZ7, S3EDD5, S3DY80, V1R5Z9, V1PR65, V1PI18, V1QYP9, V1RJP2, V1PY89, V1HZH4, V0JBC8, V1GH63, H0NE45, V1T1C1, V0GLU3, V1RYK9, S5V6G5, G5QRI8, V1NT42, V0JYQ0, V1N1G3, V4G8N5, B5C4A7, V1GBM6, V1WFX9, V0IML5, V0KGV3, V1UMB1, V1MTM7, V1MU72, V1M767, V0QHT2, H5VM16, V1LMU8, V1PN15, V1MK67, V2PLP6, V1L3F3, V1LNW1, V1LR68, V1J6D8, H6NWU6, N0C7X4, U6YQ67, U6Y9V9, U6YXU8, H8LYI1, V1FFI7, U6VHZ1, U6WP73, U6W581, U6V9H7, T2KBW7, U4ML79, V1JCN4, V1GD81, K8UFE3, K8TAZ4, K8T803, K8VSZ6, K8U1L1, K8RU58, K8T7C6, U3SKY0, S5HPB1, G5RME9, V1JUE0, G5S2P0, V1IED6, B5Q6U5, G5SID3, E9A816, B5PBV9, V1IID6, V1HP37, V1HSY3, V1FIA3, B5R5D4, B5RGH7, B4T994, B4SXB2, B5BHY4, Q5PBZ5, A9MVK3, C0Q1U3, B4TZV2, Q8XGG5, D0ZLI3, C9X7Y9, Q7CPH8, E1WDD0, U2LW04, S5EQ29, Q8KRM2, U1VVG8, L7ZS71, D4DZ87, S0ANL2, L0VU88, I3AN96, S4YPW8, A8GLB9, G0C7Z8, V5C9Q1, I0QQV9, R4I0D2, E9CL06, F3W423, I6DKW5, F3WPI8, I6DS71, B2U5C8, Q31V12, F3VDK9, E2X4U1, E2XH11, I6FHR8, Q329P5, P0AG89, I6FL72, I6HIF2, K0WTK2, F5QCS1, I6B923, F5R5G6, E3Y7I9, F5QR64, I0VJ45, I6B5V8, F5MV63, F5NNG4, F5PIK1, I6CBZ6, I6CKI2, F5PYA1, Q0SYD5, D2AAI8, F5N9G0, Q3YVX4, I6E6X9, I6F211, I2BEG0, Q2NQW5, D3V706, N1L447, N1KUQ4, N1KNB3, N1K7Y8, L0RT69, A1JHY4, E7AYK0, F0L031, R9FGW2, R9FJ06, R9FU08, F4MTY3, Q8ZJM7, D0JCD5, A4TSB9, D5B8I5, U7EX38, U7EQD0, U7FBI1, G0JDL7, B0HYY1, B0GDS7, B0HAG1, C4H054, A9Z460, A9R690, Q1C285, Q1CD20, E8P5B4, K8Q7Q1, D1TXM6, C4HQ84, I7NDX5, I7NDY9, I7XUV7, I7NH62, I7S9Y9, I7P9K6, I7ZNZ8, I7ZRD9, I8ALP1, I6L0F8, I6KBE9, I8HBE4, I7YW53, I7PFM0, I8HR09, I6HWN7, I7TZI3, I7QAL4, I6IAP4, I8CV83, I7V6P1, I7UQ87, I6JDC1, I6JG47, I7QMW6, I7WDI6, I8FXH1, I7S2Q8, I7WQI1, I7Y2F7, I7SM00, I8IM94, I6K987, I7TJP4, I8AN26, I7TZT6, I8BLE6, I8BU37, I7UWM4, I8MP34, I7VE92, I7VID0, I8EHU6, I7W3C0, I7W8V5, I8FNM1, I7WAI5, I8RGW7, U7ENK9, Q66FQ8, Q66GB9, B2JYQ0, A7FCV1, B1JQV6, B6IYY4, B6J4L4, A9KF84, A9N957, Q83BI9, A9ZJH0, G9EN44, D3HPU5, A5IEA6, Q5WUE1, Q5X2Y1, D5T7I0, U1Z621, U1S2X0, Q5ZT57, G8UZ96, M4SAV9, G3IQY0, Q604K2, V5BUC0, H8GKA5, F9ZZR1, Q0VM78, K0CB32, L0WH21, K2GXD2, B4WYG4, U7FY82, U7HJ76, C7R8D8, Q2SMA3, Q1R1J5, S2KER8, G9EB17, T2L5V4, H0J6H8, G4FA37, U7P932, C8KYG1, C5S127, B0BRA2, A3N2F7, B3GYJ0, E0EA02, E0F5Z2, E0FCC8, D9P6C4, E0EFX0, E0EM17, D9PER9, E0EZX0, A6VLB7, G8MVT1, G3ZH84, G3Z9D4, H0KDG3, L8UFP7, G3ZV07, G4AYY3, G4AW65, L8U281, C9R1N0, L8TXH2, G4B8I8, G4A9H9, G4AHF5, G4AQI1, C6AJZ4, G5G7Z4, E6L0L5, U1S9Z7, S6E9B4, Q65QD9, U1IA17, F2BZW1, Q7VN99, I3DSG0, F9GL97, F9GMR7, F9GVQ7, F9H3M5, E1XB46, Q4QMF4, P44853, A4N7E6, A4N7E7, A5UDY2, A5UHP8, A5UHP9, E3GSU3, E4QX84, A4MWZ1, A4NWW1, A4NWW8, C4EYS9, E7A6F0, D1NEZ3, T2BHH3, C9MEN5, C9MEN6, A4NF15, A4NPD2, A4N2X7, A4N2Y4, C9MFD5, C9MFD6, I3DA37, E1W2Z3, F0EQZ5, I2J8V1, I2NQA6, U4SRM8, T2RIT8, U4SF69, U4SKR5, N1V812, U4SB70, U4SBJ1, U4RP05, B8F6T7, U4RTB0, R9XRT9, F9Q8E7, H1LL97, Q0I0W6, B0UUY0, S5FCB4, T0A4P4, S9YBF5, S9YDU0, M9X284, M2VSI8, E2P7Z8, E2NYC2, M4XJ88, M4XWT3, S5P6E0, Q9CL16, S3GI59, S2KZB4, S3FQB2, G7SSX1, S3G7Z6, S3FSR7, S3H5N6, K0YRT7, K0YE44, V4N9N4, F7TKB0, U2VWW1, E8PBG6, B7I5H3, B7H063, B2HTB2, A3M237, B0V4V4, B0VKR1, F0QNW3, F5HW29, F5IKV0, L9N3E2, K1JXW1, F5JP02, K1JX05, K1KPG4, F9ID32, M8FA35, M8EX15, M8FQY8, M8H3W5, M8GRB6, M8HLY7, M8IR35, M8IFY1, M8HM94, M8IKY8, M8JWV4, M8DQM8, M8F7B5, F9J196, M8F1Z7, M8FIV5, M8F616, J3EE97, N9KZU2, S5CV60, K6MF82, J4REI0, M4QXU6, U1U1P4, K1EDD0, J4JF09, K1ECC8, K5EEC1, K5ELT2, K1G4D4, M2Z1Q8, J0TUN2, J5IY28, K6N562, K6LTY2, K5E7S2, L9N4S0, K6N1A2, K5QUG9, N8T6N2, N8SVI4, N8RE49, N8TK47, N8YZ92, N9GEN8, N9IMJ8, N9H3J7, S3T1X1, N9IHR9, N8XWH1, N9HQ93, N8UDI8, N9HLI1, N9K048, N9KDQ0, K5DUU2, J4KG27, K6NWE2, K6LN33, K5NXK3, K6K739, J0TFH7, J1B211, K5PZV5, J0SS18, K0H2W9, K9CCT9, K9AAL3, K9C169, K9BQ02, K9BSC6, K1FBI7, K6N892, L9MAW4, K2JLT9, K2IVD4, N9B8X7, N9CZS5, N9CE60, N9ATC7, V2UK15, F0KMS3, N9DBV7, R8XX81, N9F8L4, N8PH96, D0S5J9, N8Y676, S7XW95, N8RZN8, N8YIY9, S3NSM3, S3YH49, V2TFR6, D4XMH6, N9GRI5, V2U772, N8RWE0, D0SGD5, N9B1V0, N9C9C8, S7Y6D1, N9H1W8, N9HCT5, V2R903, N8TSS7, V2TQ03, N8R601, N8Z044, V2U2D9, N8RI43, U4NT73, N9EV62, N9EVT9, K6VK61, C6RN49, J4J9N4, N8Z8T0, N8Y235, N9BD16, N9AH17, Q6F801, N8U1T2, N9S350, N9LYZ0, N8NWA3, N9MMZ6, C0VKN2, N8V4G4, N8VTT0, N9NTK8, N9TGY2, N8VYY9, N8XIB3, R9ASC7, N9PDY8, N9KPJ9, N8XS54, N9N8B2, N9P1M8, N8NMS2, N8R6U5, U7GUT9, G7GA55, N9P0W1, N9SSP8, S3T325, N9QFW8, N9QAZ3, N9MT86, N8QWL5, N9LJ05, N9MKI7, N9Q1F5, N9S819, N8V2P3, N8P768, N8VN39, D0BVZ1, D6JVS0, R9B4R6, V2V1A2, N9CK20, N9BMW9, N9S6E5, C8PUD2, D5VCX5, F1VSI1, F1WCY3, F1WN39, F1W9X9, F1WTK5, F1XCA9, F1XG54, L2F6T6, U4TC36, Q4FRF4, Q1Q9Y8, A5WDL8, F5SN98, C1DJD9, M9Y3C2, B3PGW2, I3IE33, Q9HU56, B7V3M1, A6VDP6, Q02EN8, U9A2I4, E3A186, U9A2W0, U9DK01, K1CBE6, M9S912, U9K9Y6, U9K8L7, U9K6H2, U8M4Z3, U9J1I0, U9JFQ8, U8LAX6, U8KGD7, U8KQC5, U8J4P3, U8J7P0, U9IPI2, U9H860, U8IAE7, U8IHN6, U8IBU2, U8GN78, U8GLE9, U8GM96, U9HDT4, U9GHD3, U9G3N3, U9G973, U9FPF3, U9EKE4, U8XHK9, U8XAA9, U8X2L6, U8WGR5, U8VJJ8, U8VDD1, U9PAU1, U9MYK9, U9ME69, U9MDI2, U8UXC1, U8U2C1, U8U094, U8SQM7, U9LFC8, U9LA53, U8TAN7, U8SRZ7, U8RTU3, U8SJ84, U8QD27, U8Q2W7, U8QG34, U8P501, U8NYT9, U8NHR4, U8MLF9, U8MYE8, U8E153, U8DS43, U8CX74, U8CNN1, U8CAE4, U8CBX1, U8BR08, T2ERA3, U9RIU5, U9BC64, U9QBQ4, U9Q1T7, U8AI89, U8B578, K1CX04, J7DAE4, V4X2Q2, I6S5S9, U1E9L5, V4PD83, U9P391, G2L1V9, U8G013, U8FBK4, U9S837, U9E077, U8F292, H3T354, H3THV0, U9R741, S0I0S9, U9CAV3, G2U747, U6AH18, S0HRF8, U6AY26, M3BR90, N4VQY7, S0HG24, U5RIS4, U5R3B8, R9ZL23, U8ZMF1, U9PLU6, N2CI44, U9A2E8, U9AU43, V4QZI5, R8ZKJ4, U5A6W5, V4V708, T5LEQ9, U8YVI0, U9BQ83, U2ZK50, U3HCG1, K2T8C3, F2KBF3, V4PXR0, I4XMQ3, J2EVD0, M4X240, Q1IG59, Q93TF4, Q3KJH6, Q4KJR5, C3K7C1, L7H3I1, U1TFE2, G8Q1J1, U7D9H0, J2EDV4, I4KZX1, K0W9R6, I4K4H0, E2XKW7, F6AHF6, A4Y074, J7TX13, U1S789, S2KMS0, M4K616, L8MGW1, H0JBP8, I7BPP7, A5WA86, B0KN08, Q88CX7, B1J2T8, L1M563, R9VC36, U2SIQ6, M7R8I9, T2HDG1, V4GZY1, F8FVI5, U7RD81, N9UF27, S6AJW0, D7HTP0, F0E912, S6J0P4, S6K9M8, S6J4F5, U7A7M4, U2B8D9, S2FV11, J3E2M6, J2MQX4, J3DV47, J3EE79, J2Z0I8, J2YVC8, J2ZLL9, J2ZQQ2, J3GPZ0, J2TGV0, J3BJN3, J2XDQ8, J3IWI5, I4N5S4, K9ND10, U5V8T6, A4VRU9, F8H5W9, H7EVJ8, S6L370, I4CMY6, I7AHH7, U3HX63, M2TMM1, L0GG86, I4KRX3, L7FSQ5, L7FX10, L7GUU8, F3H6T3, F3JG35, S6RHM7, T2QW49, S6S6K7, S6N579, S6NKJ9, S6KSJ1, S6VRN2, S6UHZ0, S6VV13, S6KWD6, S6SDE0, S6T5D0, S6X0H4, S6KMI7, F3I853, F3DH33, F3J2Y0, K2ST58, E7PFI8, F3EGI9, F3IJ06, F3F281, F3DSK3, F2ZRT0, Q48C90, Q4ZLR3, L8N9V1, S3MR03, Q87UH3, E2MK41, F4BHD2, F4BAL4, F4BFC8, I2AYG1, E2MPY8, E2MRD5, A7JNH4, A7JPI0, A7JFJ6, A7JJC0, B0TW18, B0TX98, C6YTD1, C6YTZ9, F8GB48, A7N938, A7N9Y5, Q2A5B6, Q2A630, Q0BNM8, Q0BPA9, A4KP47, A4KPS5, K7X2L3, A7YR39, K0E5U7, K0E753, B2SED4, B2SEQ5, A0Q467, A0Q810, Q14FQ2, Q14GB0, D2AKE0, Q5NE99, Q5NEV7, A4IXL1, A4J0C0, R0H6T4, R0J3W8, M5UAY1, M5UE03, K8YAL1, R0GXL4, R0GXT2, R0GZL2, K5WCU4, K5Y0I0, K5Y3B1, K5X9F7, K5YFN4, K5WCS9, K5XVF5, A7JEB8, C6YR25, C6YS33, H6LYZ7, H6M062, H6LX60, K0BYS3, S2UD55, S5TC45, F5SZ36, M7NUV6, I1XFM0, I1YJQ8, Q31E80, I3CGA1, I3CKI1, A7C6W6, I3BT91, B6EN68, Q5E2A2, B5FBT9, H1R1V5, D0I3Y1, R1GZT4, Q1ZLP8, F2PBE5, Q6LVL0, Q1YWC4, L8J933, Q2BXL7, T1XXA2, C2HXI7, Q1V4R4, F7YJE5, U3AU75, E8LXX8, A7MX74, A6AU65, E3BIN0, C2C5W7, A2P6Z5, A1F740, A6ADI5, A6XUA9, A3GXB1, F9C248, C2JEW5, C6RVM2, J1FWL0, K5IRL5, K5KCM9, K2VDX5, J1BQQ0, K2W9H2, J1CR62, K2W8H8, J1WAB8, J1G2J2, J1L7M8, D0HV35, F9BQ32, G6Z9N8, K5RKI9, K5JQH7, G6ZVR5, G7A6A1, G7AFX8, G7AS93, G7BAP5, U7E4G5, F9CAD3, K2SX11, F8Z225, K2T8E8, K5T0X2, J1FS15, G7BZJ0, F8ZN94, K2TXX2, K2WH49, K2UEY0, K2V5J1, K5TBC0, K5M0Q7, K2V3Z3, J1NAG3, K2VGM6, J1EUH0, K5N3J6, K5SX86, K5MFA8, G7C9N6, K5N4H5, K5U6P9, L8QI60, L8QY09, L8RFR2, K5TVW6, F8ZYX2, L8RWB9, L8S3F3, K5NHG8, L8SBX0, L8SPI0, L8T952, K2WE07, K2XPS5, J1XZL2, F9AUQ9, K5NNX9, K5P836, F9BEI1, D7HJN1, D7HQY7, C6YKV8, A6A161, A3GSC3, M7FDE7, M7F276, G7TR07, M7G184, M7GJN7, M7GHW6, M7GLL4, M7HTB2, M7I2B1, M7K1F3, M7KN83, M7M301, M7JPX6, M7KLI5, M7JGZ6, M7LN17, M7LQI8, M7K680, L1R096, D0HA04, D7HFT0, C2IE11, Q9KNS8, A5F4Y6, C3LRW4, C3NUV9, C9P0B1, U0FYR6, U3B650, S7IH23, S7I4W1, F0LRW5, C9PJY9, V5FEK7, D0XDS7, M7RG25, F9S8H7, C9P1Z3, U4ZQV5, U4ZR28, M5NHL5, D0GQ47, D0HIR0, D2YLC5, D2YEC1, S6L7A1, F9TPV8, U4H420, U4EJF2, U4FDG5, U4GMH9, U4JDB8, U4JXU2, C9QNI5, F3RTX7, T5ISQ9, E1DHU5, A6B2W9, E1DCX7, L0HZR2, E1E9I2, T5FCK4, S5JF60, S5IHN1, E1D0W6, Q87KZ3, T5EYW0, T5EYE1, T5JHZ3, T5GFR2, F9RKI8, B8KBB1, A8T8M4, A8T8M5, K5SML1, K5U8D2, K5UKI8, K5ULY7, K5UZS1, A3Y073, F9RDN0, B7VHM6, A3UQV3, F9SJ80, Q8DCW3, E8VRR8, Q7MGY8, I8TD24, H8L0J0, G7UUZ9, E6WP68, I4VRC2, I4W903, I4WJN2, I4VQZ0, I4WCN7, B2FHD7, B4SSF8, M5TFA5, I0KI38, T5KI42, M5CSU8, M5CTF7, B8L3D3, L8XY66, D2UGP5, Q8PQV0, G2LYY6, K8GB31, H1XIZ2, Q4V073, Q8PDY1, B0RLW8, Q3BZ76, H8FIT3, D4TB42, D4SZ91, U4M5J6, Q5GV22, Q2NYA6, B2SKA9, G7TKN7, F1W5G4, L7H1B7, K8Z0S3, L0T3D3, Q9PCH8, B0U2T9, B2I5B5, Q87CK2, F7N9Y2, U2BRH7, Q0EZ21, H0UMH4, U2UHV8 |
| --- |
