# Supplementary material for: Large-Scale Evolutionary Analyses on SecB Subunits of Bacterial Sec System
Source: PLoS One. 2015 Mar 16;10(3):e0120417. doi: 10.1371/journal.pone.0120417 (PMC4361572; doi:10.1371/journal.pone.0120417)
Supplement: S2 Table — (DOC) [file pone.0120417.s002.doc]

Table S2 taxonomic ranges that the 3813 SecB protein sequences cover in this study

| Phylum |  | Class |  | Order |  | Family |  | Genus |  |
| --- | --- | --- | --- | --- | --- | --- | --- | --- | --- |
| Bacteroidetes  Firmicutes  Proteobacteria  Synergistetes | 9  75  3727  2 | Bacteroidia  Cytophagia  Sphingobacteriia  Bacilli  Clostridia  Alphaproteobacteria  Betaproteobacteria  Deltaproteobacteria  Gammaproteobacteria  Zetaproteobacteria  Synergistia | 4  2  3  69  6  549  330  7  2840  1  2 | Bacteroidales  Cytophagales  Sphingobacteriales  Bacillales  Lactobacillales  Clostridiales  Thermoanaerobacterales  Caulobacterales  Magnetococcales  Parvularculales  Rhizobiales  Rhodobacterales  Rhodospirillales  Rickettsiales  Sphingomonadales  Burkholderiales  Gallionellales  Hydrogenophilales  Methylophilales  Neisseriales  Nitrosomonadales  Rhodocyclales  Desulfobacterales  Desulfovibrionales  Desulfuromonadales  Acidithiobacillales  Aeromonadales  Alteromonadales  Cardiobacteriales  Chromatiales  Enterobacteriales  Legionellales  Methylococcales  Oceanospirillales  Pasteurellales  Pseudomonadales  Thiotrichales  Vibrionales  Xanthomonadales  Mariprofundales  Synergistales | 4  2  3  1  68  4  2  17  1  1  320  55  56  72  27  148  2  2  3  160  3  12  4  1  2  5  17  75  3  22  1835  17  5  16  112  431  71  186  45  1  2 | Bacteroidaceae  Porphyromonadaceae  Prevotellaceae  Cyclobacteriaceae  Saprospiraceae  Paenibacillaceae  Enterococcaceae  Lactobacillaceae  Streptococcaceae  Clostridiaceae  Lachnospiraceae  Thermoanaerobacteraceae  Thermoanaerobacterales Family III Incertae Sedis  Caulobacteraceae  Magnetococcaceae  Parvularculaceae  Aurantimonadaceae  Bartonellaceae  Beijerinckiaceae  Bradyrhizobiaceae  Brucellaceae  Hyphomicrobiaceae  Methylobacteriaceae  Methylocystaceae  Phyllobacteriaceae  Rhizobiaceae  Xanthobacteraceae  Hyphomonadaceae  Rhodobacteraceae  Acetobacteraceae  Rhodospirillaceae  Anaplasmataceae  Candidatus Midichloriaceae  Rickettsiaceae  Erythrobacteraceae  Sphingomonadaceae  Alcaligenaceae  Burkholderiaceae  Comamonadaceae  Oxalobacteraceae  Sutterellaceae  Gallionellaceae  Hydrogenophilaceae  Methylophilaceae  Neisseriaceae  Nitrosomonadaceae  Rhodocyclaceae  Desulfobulbaceae  Desulfovibrionaceae  Geobacteraceae  Acidithiobacillaceae  Aeromonadaceae  Succinivibrionaceae  Alteromonadaceae  Colwelliaceae  Ferrimonadaceae  Idiomarinaceae  Pseudoalteromonadaceae  Psychromonadaceae  Shewanellaceae  Cardiobacteriaceae  Chromatiaceae  Ectothiorhodospiraceae  Halothiobacillaceae  Enterobacteriaceae  Coxiellaceae  Legionellaceae  Methylococcaceae  Alcanivoracaceae  Hahellaceae  Halomonadaceae  Pasteurellaceae  Moraxellaceae  Pseudomonadaceae  Francisellaceae  Piscirickettsiaceae  Thiotrichaceae  Vibrionaceae  Sinobacteraceae  Xanthomonadaceae  Mariprofundaceae  Synergistaceae | 2  1  1  2  3  1  2  4  62  2  2  1  1  17  1  1  1  23  2  27  198  3  11  1  10  41  3  3  52  43  13  28  1  43  2  25  20  85  24  10  9  2  2  3  160  3  12  4  1  2  5  15  2  28  1  1  3  15  1  26  3  13  8  1  1835  6  11  5  8  1  7  112  196  235  59  8  4  186  1  44  1  2 | Bacteroides  Paludibacter  Prevotella  Belliella  Cyclobacterium  Saprospira  Paenibacillus  Enterococcus  Lactobacillus  Streptococcus  Clostridium  Blautia  Moorella  Thermoanaerobacterium  Asticcacaulis  Brevundimonas  Caulobacter  Phenylobacterium  Magnetococcus  Parvularcula  Aurantimonas  Bartonella  Beijerinckia  Methylocella  Afipia  Bradyrhizobium  Nitrobacter  Oligotropha  Rhodopseudomonas  Brucella  Ochrobactrum  Hyphomicrobium  Pelagibacterium  Rhodomicrobium  Methylobacterium  Microvirga  Methylocystis  Chelativorans  Mesorhizobium  Nitratireductor  Agrobacterium  Candidatus Liberibacter  Rhizobium  Shinella  Sinorhizobium  Azorhizobium  Starkeya  Xanthobacter  Hyphomonas  Maricaulis  Oceanicaulis  Celeribacter  Citreicella  Dinoroseobacter  Jannaschia  Ketogulonicigenium  Labrenzia  Loktanella  Maritimibacter  Oceanibulbus  Oceanicola  Oceaniovalibus  Octadecabacter  Paracoccus  Pelagibaca  Phaeobacter  Pseudovibrio  Rhodobacter  Rhodovulum  Roseibium  Roseobacter  Roseovarius  Rubellimicrobium  Ruegeria  Sagittula  Salipiger  Sulfitobacter  Thalassobacter  Thalassobium  Acetobacter  Acidiphilium  Gluconacetobacter  Gluconobacter  Granulibacter  Roseomonas  Azospirillum  Caenispirillum  Magnetospirillum  Oceanibaculum  Phaeospirillum  Rhodospirillum  Thalassospira  Anaplasma  Ehrlichia  Neorickettsia  Wolbachia  Candidatus Midichloria  Orientia  Rickettsia  Erythrobacter  Novosphingobium  Sphingobium  Sphingomonas  Sphingopyxis  Zymomonas  Achromobacter  Alcaligenes  Bordetella  Pusillimonas  Taylorella  Burkholderia  Cupriavidus  Lautropia  Limnobacter  Ralstonia  Acidovorax  Albidiferax  Alicycliphilus  Comamonas  Delftia  Hylemonella  Polaromonas  Ramlibacter  Variovorax  Verminephrobacter  Herbaspirillum  Herminiimonas  Janthinobacterium  Massilia  Oxalobacter  Parasutterella  Sutterella  Gallionella  Sideroxydans  Sulfuricella  Thiobacillus  Methylobacillus  Methylotenera  Chromobacterium  Kingella  Laribacter  Neisseria  Pseudogulbenkiania  Nitrosomonas  Nitrosospira  Aromatoleum  Azoarcus  Dechloromonas  Methyloversatilis  Thauera  Desulfobulbus  Desulfocapsa  Desulfotalea  Desulfurivibrio  Desulfovibrio  Geobacter  Acidithiobacillus  Aeromonas  Oceanimonas  Tolumonas  Succinatimonas  Agarivorans  Alishewanella  Alteromonas  Glaciecola  Marinobacter  Saccharophagus  Colwellia  Ferrimonas  Idiomarina  Pseudoalteromonas  Psychromonas  Shewanella  Cardiobacterium  Dichelobacter  Allochromatium  Nitrosococcus  Rheinheimera  Thiocapsa  Thiocystis  Thioflavicoccus  Thiorhodococcus  Thiorhodovibrio  Alkalilimnicola  Ectothiorhodospira  Halorhodospira  Spiribacter  Thioalkalivibrio  Thiorhodospira  Halothiobacillus  Arsenophonus  Brenneria  Buchnera  Candidatus Blochmannia  Candidatus Hamiltonella  Candidatus Regiella  Cedecea  Citrobacter  Cronobacter  Dickeya  Edwardsiella  Enterobacter  Erwinia  Escherichia  Hafnia  Klebsiella  Kosakonia  Pantoea  Pectobacterium  Photorhabdus  Plesiomonas  Proteus  Providencia  Rahnella  Salmonella  Serratia  Shigella  Shimwellia  Sodalis  Xenorhabdus  Yersinia  Coxiella  Legionella  Methylobacter  Methylococcus  Methyloglobulus  Methylomicrobium  Methylomonas  Alcanivorax  Kangiella  Hahella  Chromohalobacter  Halomonas  Actinobacillus  Aggregatibacter  Avibacterium  Basfia  Gallibacterium  Haemophilus  Histophilus  Mannheimia  Pasteurella  Acinetobacter  Enhydrobacter  Moraxella  Psychrobacter  Azotobacter  Cellvibrio  Pseudomonas  Francisella  Cycloclasticus  Methylophaga  Thiomicrospira  Beggiatoa  Thiothrix  Aliivibrio  Grimontia  Photobacterium  Vibrio  Hydrocarboniphaga  Frateuria  Pseudoxanthomonas  Rhodanobacter  Stenotrophomonas  Wohlfahrtiimonas  Xanthomonas  Xylella  Mariprofundus  Jonquetella | 2  1  1  1  1  3  1  2  4  62  2  2  1  1  6  5  5  1  1  1  1  23  1  1  2  15  2  1  7  193  5  1  1  1  10  1  1  1  7  2  8  4  20  1  8  1  1  1  1  1  1  1  2  1  1  2  3  1  1  1  1  1  1  2  1  1  2  7  1  1  6  3  1  6  1  1  1  1  1  27  3  3  8  1  1  3  1  2  1  1  4  1  10  5  2  11  1  2  41  2  7  8  5  2  3  6  1  9  1  3  64  6  1  1  13  7  1  1  2  4  1  4  1  2  1  4  1  2  1  2  1  8  1  1  1  1  1  2  1  4  1  152  2  2  1  1  1  1  1  8  1  1  1  1  1  2  5  13  1  1  2  1  2  8  7  9  1  1  1  3  15  1  26  2  1  1  4  2  1  1  1  1  2  1  1  1  1  3  1  1  1  1  10  1  1  2  1  11  10  3  6  27  13  999  1  122  1  12  6  4  1  4  9  1  449  16  34  1  1  1  86  6  11  1  1  1  1  1  7  1  1  1  6  14  19  1  1  1  50  2  11  13  181  1  9  5  2  2  231  59  3  4  1  3  1  4  2  6  174  1  1  2  5  8  1  21  6  1  2 |
